# Assessing the Implementation of Digital Innovations in Response to the COVID-19 Pandemic to Address Key Public Health Functions: Scoping Review of Academic and Nonacademic Literature

**DOI:** 10.2196/34605

**Published:** 2022-07-06

**Authors:** Joseph Francombe, Gemma-Claire Ali, Emily Ryen Gloinson, Carolina Feijao, Katherine I Morley, Salil Gunashekar, Helena de Carvalho Gomes

**Affiliations:** 1 RAND Europe Cambridge United Kingdom; 2 European Centre for Disease Prevention and Control Stockholm Sweden

**Keywords:** digital technologies, COVID-19, key public health functions, scoping review, digital health, pandemic, surveillance, mobile phone

## Abstract

**Background:**

Digital technologies have been central to efforts to respond to the COVID-19 pandemic. In this context, a range of literature has reported on developments regarding the implementation of new digital technologies for COVID-19–related surveillance, prevention, and control.

**Objective:**

In this study, scoping reviews of academic and nonacademic literature were undertaken to obtain an overview of the evidence regarding digital innovations implemented to address key public health functions in the context of the COVID-19 pandemic. This study aimed to expand on the work of existing reviews by drawing on additional data sources (including nonacademic sources) by considering literature published over a longer time frame and analyzing data in terms of the number of unique *digital innovations*.

**Methods:**

We conducted a scoping review of the academic literature published between January 1, 2020, and September 15, 2020, supplemented by a further scoping review of selected nonacademic literature published between January 1, 2020, and October 13, 2020. Both reviews followed the PRISMA (Preferred Reporting Items for Systematic Reviews and Meta-Analyses) approach.

**Results:**

A total of 226 academic articles and 406 nonacademic articles were included. The included articles provided evidence of 561 (academic literature) and 497 (nonacademic literature) unique digital innovations. The most common implementation settings for digital innovations were the United States, China, India, and the United Kingdom. Technologies most commonly used by digital innovations were those belonging to the high-level technology group of *integrated and ubiquitous fixed and mobile networks*. The key public health functions most commonly addressed by digital innovations were communication and collaboration and surveillance and monitoring.

**Conclusions:**

Digital innovations implemented in response to the COVID-19 pandemic have been wide ranging in terms of their implementation settings, the digital technologies used, and the public health functions addressed. However, evidence gathered through this study also points to a range of barriers that have affected the successful implementation of digital technologies for public health functions. It is also evident that many digital innovations implemented in response to the COVID-19 pandemic are yet to be formally evaluated or assessed.

## Introduction

### Background

Digital technologies, such as artificial intelligence (AI), robotics, and wearables, have been widely used in worldwide efforts to respond to the COVID-19 pandemic. In this context, a range of studies has reported on developments regarding the implementation of new digital technologies for COVID-19–related surveillance, prevention, and control. To consolidate this literature, several reviews have been undertaken [1-4]. Broadly, the aim of these reviews has been to describe the characteristics of digital technologies that have been reported on within the early scientific literature. Golinelli et al [2] searched MEDLINE and medRxiv to identify the relevant literature on the use of digital technologies in health care during the COVID-19 pandemic. The included papers were then analyzed in terms of article characteristics and the type of technology and *patient needs addressed*. A review conducted by Budd et al [1] provided a qualitative overview of the breadth of digital innovations introduced as part of the global public health response to the COVID-19 pandemic, the types of public health activities they addressed, and the key potential barriers to their implementation. Vargo et al [4] further reviewed digital technology use during the COVID-19 pandemic based on searches of 4 databases: Web of Science, Scopus, PubMed, and Google Scholar. The review synthesized the evidence from included papers in relation to 4 key areas of *technologies*, *users*, *activities*, and *effects* within the spheres of *health care*, *education*, *work*, and *daily life* [4]. More recently, Mbunge et al [3] undertook a critical review of emerging technologies for tackling the COVID-19 pandemic, focusing on prevention, surveillance, and containment, based on searches of the following sources: Google Scholar, Scopus, ScienceDirect, PubMed, IEEE Xplore Digital Library, ACM Digital Library, Wiley Library, and SpringerLink [3]. Although providing valuable overviews of the digital response to the COVID-19 pandemic, existing reviews have also been limited by a focus on academic sources, thereby potentially missing developments reported in wider nonacademic literature while also tending to focus on the early period of the pandemic.

### Study Aims

In this study, we present the findings of a further scoping review on the implementation of digital technologies in response to the COVID-19 pandemic. The scoping review expands on the work of existing reviews by drawing on additional data sources while also considering literature published over a longer time frame (ie, January to September 2020). Although focusing on academic literature, the scoping review also goes beyond existing reviews by presenting evidence from a complementary review of nonacademic sources, including web-based technology-related news sources and news feeds (covering news articles, press releases, and blogs). The incorporation of wider nonacademic sources into this review allows for the consideration of technological developments in the private or public sector, which are not necessarily oriented toward research publications, thus helping to capture more up-to-date information on the implementation of digital technologies in response to the COVID-19 pandemic.

This scoping review goes beyond existing reviews by using the concept of *digital innovations*. By digital innovations, we refer to the application ≥1 digital technology to address COVID-19–related key public health functions within a single application in a specific context. An example of a digital innovation captured by this scoping review is Austria’s contact-tracing app *Stopp Corona*. The app combines 2 digital technologies of interest in this review—smartphone apps and Bluetooth—into a single digital innovation [5,6]. By analyzing data regarding the *number of implemented digital innovations* and their characteristics, this study goes another step beyond existing reviews, all of which have analyzed digital technology trends by considering the *number of papers* reporting on different technology types and functions [1-4]

The specific research questions addressed by this scoping review were as follows:

1. What are the main characteristics of the literature reporting on digital innovations used in the context of the COVID-19 pandemic?
2. What has been the geographical setting of the digital innovations implemented in response to the COVID-19 pandemic?
3. What types of implemented digital innovations have been discussed in the academic and nonacademic literature in relation to COVID-19–related surveillance, prevention, and control?
4. Which key public health functions have been addressed by the digital innovations implemented in response to the COVID-19 pandemic?

## Methods

### Overview

The scoping review followed the approach specified in PRISMA-ScR (Preferred Reporting Items for Systematic Reviews and Meta-Analyses extension for Scoping Reviews) checklist [7]. A completed PRISMA-ScR checklist for the review is presented in Multimedia Appendix 1. A study protocol was developed presenting key elements of the proposed approach and methods to be used. The approach comprised 2 parallel methodological approaches—one for the review of academic literature and the other for the review of nonacademic literature.

### Search Strategy

For the academic literature search, we developed and ran a search strategy in 2 bibliographic databases—EMBASE and Scopus—using the same strategy for both databases. The search terms used in this study are presented in Multimedia Appendix 2. The search was limited to articles published between January 1 and September 15, 2020 (the date of the search), and included English-language and non–English-language articles. The search strategy drew on a strategy developed for a previous scoping review on digital technologies for infectious disease surveillance, prevention, and control, which was peer-reviewed using the Peer-Review of Electronic Search Strategies approach [8]. In addition to the database searches, we also conducted targeted searches using Google Scholar to identify a small number of additional academic articles where the results of the database search strategies revealed evidence gaps.

For the nonacademic literature search, we ran a search strategy using the news aggregation software Feedly [9]. To conduct the Feedly search, we identified relevant information sources covering digital technological innovation and health innovation based on expert consultation and internal piloting. The search strategy applied to these information sources was broadly aligned with that used for the academic literature search. However, because of the limitations of the Feedly search function, the search string used was shorter and more generic than that used for the academic search. The used search terms are presented in Multimedia Appendix 2. The Feedly search traced backward to capture articles published between January 1 and September 15, 2020 (the date of the initial search), but also captured articles published over a period of 4 weeks from the initial search date (ie, between September 15 and October 13, 2020). To manage the scope of the study, the nonacademic literature search was limited to English-language sources only. As with the academic literature search, we also conducted targeted searches to identify a small number of additional nonacademic articles. In addition to a systematic Google search, targeted searching of nonacademic literature included scraping selected websites to identify relevant articles. The selection of websites focused on addressing gaps in the evidence produced by the Feedly-based searches, specifically the limited number of articles reporting on developments within the European Union or European Economic Area (EU/EEA) region compared with those reporting on technological developments outside the EU or EEA.

### Study Selection

Articles captured by both searches were screened against defined inclusion and exclusion criteria to determine their eligibility for the study. Multimedia Appendix 3 presents the used inclusion and exclusion criteria. In the review of academic literature, non-English articles were included in the study selection but only if an English-language abstract or summary was available. Screening was undertaken by 2 study teams, each comprising 2 researchers—one study team for the review of academic literature and one study team for the review of nonacademic literature. Before commencing the study selection, both study teams engaged in pilot screening exercises for 100 articles to ensure consistency in the application of the eligibility criteria. The 2 reviewers discussed the areas of uncertainty or disagreement until full agreement on inclusion or exclusion was reached. To further ensure consistency across the study teams, we held weekly cross-project meetings. During these meetings, any articles for which a reviewer was unsure were marked and discussed with the other study team to determine inclusion or exclusion. A shared log of the inclusion and exclusion decisions was maintained across the 2 study teams.

### Data Extraction

We extracted data from the included articles using Microsoft Excel extraction templates: one template for the review of academic literature and one for the review of nonacademic literature. Both extraction templates included columns to capture information relating to the core research questions regarding the types and nature of implemented digital innovations, as well as broader information regarding the article type and identified barriers to implementing digital innovations in the discussed countries and regions. Where possible, drop-down menus were used to limit the range of responses that could be submitted, thereby facilitating data filtering and analysis. To ensure a consistent extraction approach, the 2 project teams conducted pilot extraction exercises using a small number of articles. The used extraction templates and drop-down menus are presented in Multimedia Appendix 4.

### Data Analysis

To analyze the extracted data, we used the software package R. Descriptive quantitative analysis focused on statistical and graphical summaries for each column of data captured using drop-down menus in the extraction template, together with relevant cross-analyses. Data extracted on barriers to the implementation of digital innovations were analyzed qualitatively.

### Key Study Variables

#### High-Level Technology Groups and Specific Digital Technologies

In extracting data on digital innovations, we coded data on the specific digital technologies that have been used within these innovations, as well as the technology groups to which these technologies belong. The used coding approach drew on an earlier scoping review of the use of digital technologies for the prevention, surveillance, and control of infectious diseases. In this study, specific technologies identified in the literature were clustered into high-level technology groups of similar or conceptually related digital technologies [10]. For this study, definitions for each specific digital technology and each high-level technology group were established using the European Commission’s Digital Single Market glossary, supplemented, where necessary, by definitions from relevant academic literature [11]. The coding approach is presented in Textbox 1.

**Coding of specific digital technologies into high-level technology groups.**

**High-level technology group and specific digital technology**

- Advanced manufacturing technologies
  - 3D printing
- Autonomous devices and systems
  - Drones
  - Robotics
- Blockchain or distributed ledger technology
  - Blockchain or distributed ledger technology
- Cloud computing or cloud-based networks
  - Cloud computing or cloud-based networks
- Cognitive technologies
  - Artificial intelligence
  - Expert systems
  - Machine learning
  - Natural language processing
  - Facial recognition
  - (Artificial) neural networks
- Crowdsourcing platforms
  - Crowdsourcing
- Data analytics (including big data)
  - Data mining
  - Data analytics
  - Big data
  - Health informatics
  - Parallel computing
  - Social media and mobile data analysis
- eHealth
  - Digital health, eHealth, and mobile health
  - Electronic health records
  - Telemedicine
- Imaging and sensing technologies (including Geographic Information System)
  - Geographic Information System
  - Image processing
  - Infrared sensing
  - Satellite communication or imaging (including earth observation and remote sensing)
- Immersive technologies
  - Virtual or augmented reality
- Integrated and ubiquitous fixed and mobile networks
  - Cellular networks
  - SMS text message communications
  - Smartphone apps
  - Bluetooth
  - Smartphones and tablet computing devices
- Web-based tools and platforms
  - Web-based learning platforms
  - Web-based self-assessment tools
  - Information management tools
- Social media
  - Microblogging platforms
  - Blogging platforms
  - Instant messaging platforms
  - Networking platforms
  - Photograph or video-sharing platforms
- Internet of Things
  - Internet of Things
  - Wireless sensor networks
  - Biosensors
- Nanotechnology and microsystems
  - Digital DNA, RNA or protein analysis
  - Lab-on-chip
  - Nanotechnology
- Quantum computing
  - Quantum computing
- Simulation
  - Mathematical models or simulations
- Wearables (including ingestibles)
  - Wearables (including smart fabrics and ingestibles)

#### Key Public Health Functions

We also coded each digital innovation as fulfilling ≥1 of the following seven key public health functions: (1) screening and diagnostics, (2) surveillance and monitoring, (3) contact tracing, (4) forecasting, (5) signal or outbreak detection and validation, (6) pandemic response, and (7) communication and collaboration. The use of these public health key functions followed a mapping of the European Centre for Disease Prevention and Control’s priorities against the 10 essential public health operations of the World Health Organization’s Regional Office for Europe [12] and the US Center for Disease Control’s 10 essential public health services [13]. On the basis of this mapping exercise, the public health key functions used in this study were also refined to suit the COVID-19 context. For example, to better reflect the diverse range of activities undertaken to ensure safe access to and management of essential resources during the COVID-19 pandemic, including at the population level, *pandemic response* was included as a key public health function. Similarly, to reflect its centrality in response to the pandemic, contact tracing was also included as a distinct key public health function. Textbox 2 presents the high-level definitions of these key public health functions for the purposes of this study. The key public health functions used in this study were neither exhaustive nor definitive. For example, the used functions do not cover the application of emerging digital technologies to the development of treatments or vaccines. Other studies may adopt alternative approaches to identifying and defining key public health functions.

As with the classification of technologies, our data extraction template included columns to record instances in which a digital innovation addressed >1 key public health function. For example, digital innovations performing surveillance and monitoring functions and signal or outbreak detection and validation were coded with both these public health functions. The coding of key public health functions was based on data extracted from the academic or nonacademic sources being reviewed. The emphasis within the article guided the assessment of how the codes were applied. The collation of data from multiple sources on the same innovations allowed us to capture where digital innovations addressed >2 key public health functions.

**Key public health functions and definitions.**

**Screening and diagnostics**

- Identifying (including self-identifying) COVID-19 symptoms and the presence of SARS-CoV-2 in individuals

**Surveillance and monitoring**

- Systematic collection and analysis of relevant data such as SARS-CoV-2 infection rates and excess deaths along with ongoing monitoring of COVID-19 symptoms or adherence to COVID-19 restrictions at the individual and population levels

**Contact tracing**

- Identifying and alerting people who have been in contact with someone diagnosed with COVID-19 and who are therefore at high risk of having been exposed to SARS-CoV-2, so that they can take appropriate and sometimes mandated action (eg, self-isolating)

**Forecasting**

- Predicting COVID-19 infections or health outcomes at the individual and population levels

**Signal or outbreak detection and validation**

- Detecting and validating outbreaks of COVID-19

**Pandemic response**

- Responses to the pandemic that have helped widen safe access to and management of essential resources required by individuals and populations for COVID-19 prevention and response

**Communication and collaboration**

- Communication involves informing, educating, and empowering individuals and populations about COVID-19, and collaboration refers to working together across disciplines or sectors to share knowledge and improve the collective COVID-19 response

## Results

### Search Results

The search of academic databases returned a total of 5018 articles, of which 1408 (28.06%) were duplicates. Title and abstract screening of the remaining 3610 articles resulted in 3309 (91.66%) articles being excluded, with 301 (8.34%) deemed eligible for full-text review. Through targeted searches, we also included an additional 6 articles. Of these 307 articles, 81 (26.4%) were excluded during data extraction and analysis, resulting in 226 (73.6%) unique articles being included in the review of the academic literature.

**Figure 1:**
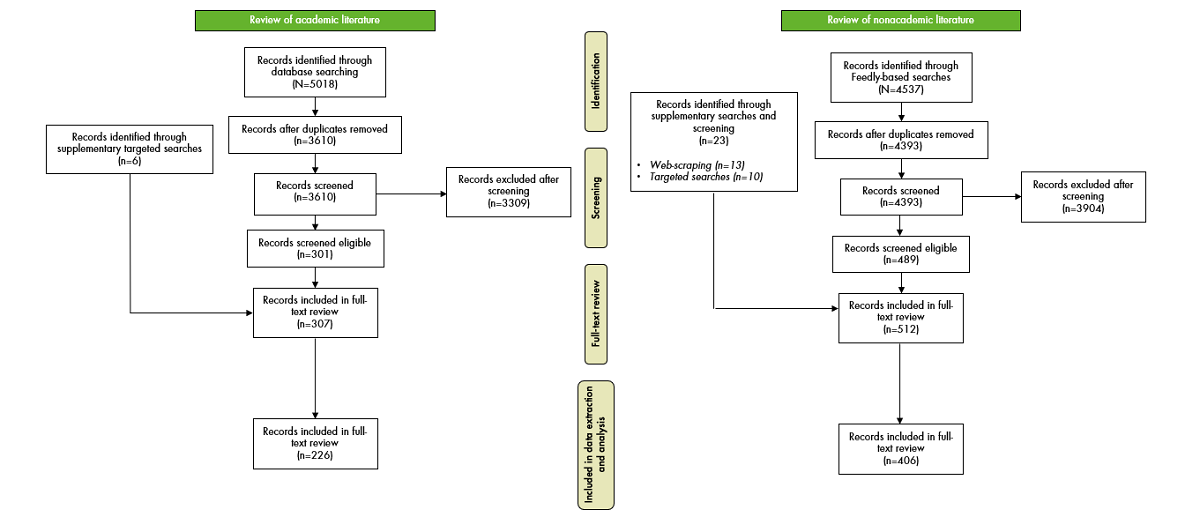
PRISMA (Preferred Reporting Items for Systematic Reviews and Meta-Analyses) flow diagrams for the review of academic literature (left) and nonacademic literature (right; review time frame for academic literature: January 1, 2020, to September 15, 2020; review time frame for nonacademic literature: January 1, 2020, to October 13, 2020).

For the review of nonacademic literature, the Feedly-based literature search returned a total of 4537 articles, of which 144 (3.17%) were duplicates. Title screening of the remaining 4393 articles resulted in 3904 (88.87%) articles being excluded, with 489 (11.13%) included for full-text review. We also included an additional 23 articles through targeted searching (n=10, 43% articles) and web scraping (n=13, 57% articles). Of these 512 articles, 107 (20.9%) were excluded during the data extraction and analysis. This resulted in 79.2% (406/512) unique nonacademic articles being included. PRISMA (Preferred Reporting Items for Systematic Reviews and Meta-Analyses) flow diagrams for the 2 scoping reviews are presented in Figure 1.

### Characteristics of the Included Literature

All articles included in the review of academic literature fell into one of the following 3 article types: brief journal comment, editorial, letter, or opinion (98/226, 43.4% of articles), research articles (86/226, 38.1% articles), and detailed journal perspective, policy review, or practice reviews (42/226, 18.6% of articles). The 86 articles categorized as research articles were further analyzed in terms of their study type, with the following results: 67 (78%) primary studies, 2 (2%) mathematical models or simulations, 2 (2%) scoping reviews, and 14 (16%) other literature reviews. The study type of one of the research articles was unclear. All articles included in the review of nonacademic literature were news articles, blog posts, or press releases.

The publication dates of the included articles by month are shown in Table 1. For the review of academic literature, most included articles (203/226, 89.8% of articles) were published in April, May, June, July, and August, with June being the month in which the highest number of included articles (46/226, 20.4% articles) were published. For the review of nonacademic literature, a higher number of included articles (250/406, 61.6%) were published in March, April, and May. For comparison, 34% (138/406) of articles were published in June, July, August, and September. Across both reviews, a small number of articles were published in February, September, and October.

For articles identified by the review of academic literature, we analyzed the geographical location of key contributors’ (first, last, and corresponding authors’) organizations. The country with the highest number of key contributor organizational affiliations was the United States (64/226, 28.3% of articles). Other countries with a high number of key contributor organizational affiliations included China (23/226, 10.2% of articles), the United Kingdom (20/226, 8.8% of articles), and India (13/226, 5.8% of articles). Table 2 presents the 10 countries with the highest number of key contributor organizational affiliations.

**Table 1:** Number of articles by publication date (by month) in the review of academic literature and nonacademic literature (review time frame for academic literature: January 1, 2020, to September 15, 2020; review time frame for nonacademic literature: January 1, 2020, to October 13, 2020)^a^.

Publication date		Included articles, n (%)
**Review of academic literature (N=226)**		
	January	0 (0)
	February	3 (1.3)
	March	10 (4.4)
	April	38 (16.8)
	May	39 (17.3)
	June	46 (20.4)
	July	39 (17.3)
	August	41 (18.1)
	September	10 (4.4)
**Review of nonacademic literature (N=406)^b^**		
	January	0 (0)
	February	6 (1.5)
	March	66 (16.3)
	April	108 (26.7)
	May	76 (18.8)
	June	36 (8.9)
	July	31 (7.7)
	August	33 (8.1)
	September	38 (9.4)
	October	11 (2.7)

^a^Due to rounding, the percentages for the nonacademic review do not add up to 100.

^b^For one article within the review of nonacademic literature, no publication date was recorded. Although 406 articles were reviewed, the articles listed against months in the table therefore add up to 405.

**Table 2:** Top 10 countries in which key contributors’ organizations were based in the review of academic literature (review time frame: January 1, 2020, to September 15, 2020; N=226).

Country of key contributors’ organization	Included articles, n (%)
United States	64 (28.3)
China (mainland)	23 (10.2)
United Kingdom	20 (8.8)
India	13 (5.8)
Australia	8 (3.5)
France	8 (3.5)
Taiwan	8 (3.5)
Singapore	7 (3.1)
South Korea	7 (3.1)
Spain	6 (2.7)

### Number of Digital Innovations Implemented in Response to the COVID-19 Pandemic

Through our review of the academic literature, we identified 561 instances of the implementation of digital innovations to tackle the COVID-19 pandemic. Our review of the nonacademic literature identified 497 digital innovations. The 2 reviews were conducted independently. As such, the digital innovations identified by the review of nonacademic literature are not necessarily presented as unique from those identified by the review of academic literature. Although there is likely to be some crossover between the innovations captured by the 2 reviews, it is also the case that the more experimental review of nonacademic literature has captured some innovations, in particular those developed by private companies, that have not been captured within the review of academic literature, particularly as developments occurred rapidly during the first few months of the pandemic in 2020.

### Geographic Context of Digital Innovations Implemented in Response to the COVID-19 Pandemic

The identified digital innovations were analyzed by the geographical context in which they were implemented at both the regional and country levels. In the academic literature, 66.5% (373/561) of digital innovations were implemented in non-EU/EEA countries, with 21.4% (120/561) of digital innovations being implemented within EU/EEA countries. Approximately 12.1% (68/561) of digital innovations identified in our review were implemented worldwide. The countries with the highest number of implemented digital innovations, according to academic literature, were the United States of America (107/561, 19.1% of digital innovations), China (71/561, 12.7% of digital innovations), and India (28/561, 5% of digital innovations). The most common EU or EEA country implementation settings were France (18/561, 3.2% of digital innovations), Spain (18/561, 3.2% of digital innovations), and Italy (12/561, 2.1% of digital innovations; see Table 3).

**Table 3:** Top 10 countries in which the highest number of digital innovations have been implemented in the review of academic literature and nonacademic literature (review time frame for academic literature: January 1, 2020, to September 15, 2020; review time frame for nonacademic literature: January 1, 2020, to October 13, 2020).

Implementation setting		Digital innovations, n (%)
**Review of academic literature (N=561)**		
	United States	107 (19.1)
	China	71 (12.7)
	India	28 (5.0)
	United Kingdom	25 (4.5)
	France	18 (3.2)
	Spain	18 (3.2)
	South Korea	16 (2.9)
	Singapore	13 (2.3)
	Canada	12 (2.1)
	Italy	12 (2.1)
**Review of nonacademic literature (N=497)**		
	United States	141 (28.4)
	United Kingdom	60 (12.1)
	China	38 (7.6)
	Italy	13 (2.6)
	Spain	11 (2.2)
	Germany	10 (2)
	Singapore	10 (2)
	France	8 (1.6)
	India	8 (1.6)
	Australia, Israel, and The Netherlands	7 (1.4)

Evidence from the nonacademic literature supports this overarching picture, with most of the identified digital innovations implemented in non-EU or EEA countries (320/497, 64.4% of digital innovations) and a smaller number of digital innovations implemented within the EU or EEA (89/497, 17.9% of digital innovations). According to the nonacademic literature, the most common implementation settings were the United States (141/497, 28.4% of digital innovations), the United Kingdom (60/497, 12.1% of digital innovations), and China (38/497, 7.6% of digital innovations), with Italy (13/497, 2.6% of digital innovations), Spain (11/497, 2.2% of digital innovations), Germany (10/497, 2% of innovations), and France (8/497, 1.6% of digital innovations) being the most common EU or EEA country implementation settings (Table 3).

### Types of Digital Innovations Implemented in Response to the COVID-19 Pandemic

The digital innovations were also analyzed in terms of the types of digital technology they incorporated. As explained previously, we analyzed innovations in terms of the specific digital technologies they incorporated and the high-level technology groups to which these specific technologies belonged.

**Figure 2:**
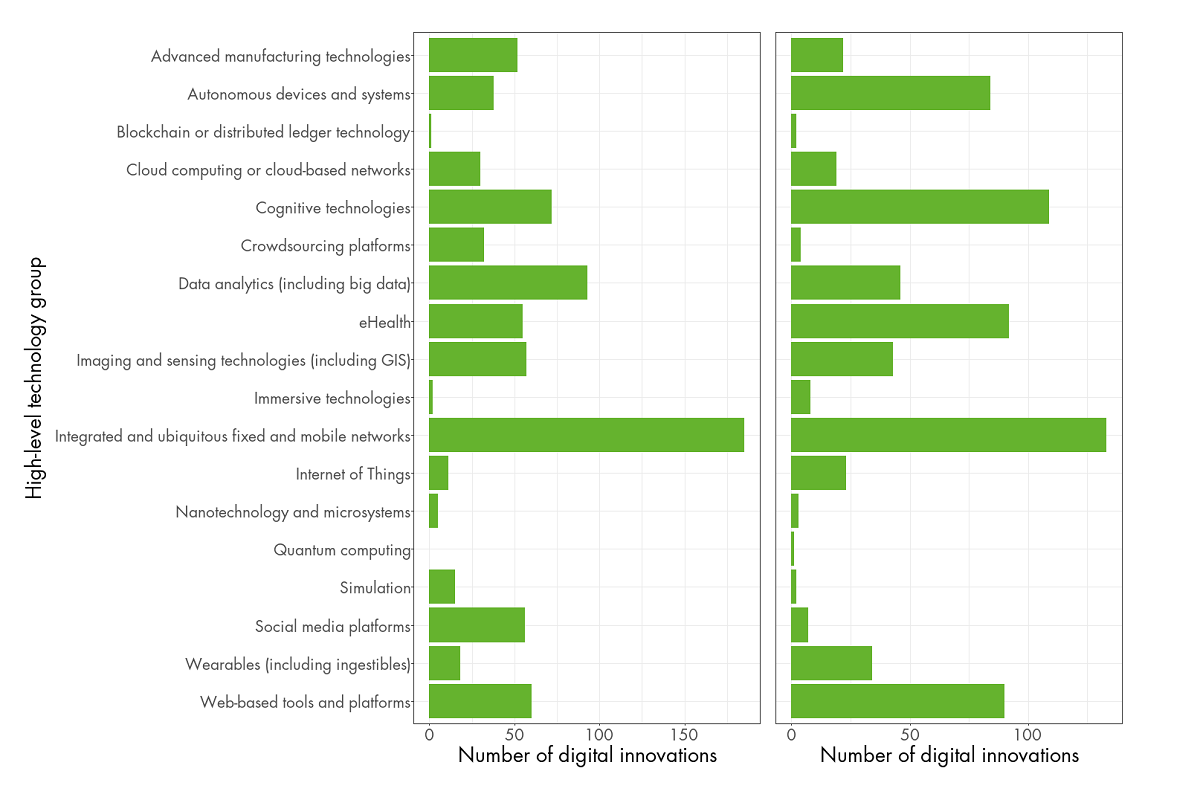
Number of digital innovations using each high-level technology group in the review of academic literature (left) and nonacademic literature (right; review time frame for academic literature: January 1, 2020, to September 15, 2020; review time frame for nonacademic literature: January 1, 2020, to October 13, 2020). GIS: Geographic Information Systems.

In the academic literature, the most commonly implemented high-level technology group was *integrated and ubiquitous fixed and mobile networks* (185/561, 33% of digital innovations incorporated at least one digital technology falling under this high-level group; Figure 2, left frame). Other high-level technology groups with a high number of digital innovations were *data analytics* (93/561, 16.6% of digital innovations), *cognitive technologies* (72/561, 12.8% of digital innovations), and *web-based tools and platforms* (60/561, 10.7% of digital innovations). The most commonly used specific digital technology was smartphone apps (144/561, 25.7% of all digital innovations and 144/185, 77.8% of digital innovations within the *integrated and ubiquitous fixed and mobile networks* high-level technology group).

In the nonacademic literature, *integrated and ubiquitous fixed and mobile networks* were also the most commonly implemented high-level technology group (133/497, 26.8% of digital innovations; Figure 2, right frame). Other high-level technology groups with a high number of digital innovations were *cognitive technologies* (109/497, 21.9% of digital innovations), *eHealth* (92/497, 18.5% of digital innovations), and *web-based tools and platforms* (90/497, 18.1% of digital innovations). The most commonly included specific digital technology, according to the nonacademic literature, was AI (90/497, 18.1% of all digital innovations and 90/109, 82.6% of digital innovations within the *cognitive technologies* high-level technology group).

### Key Public Health Functions Addressed by Digital Innovations Implemented in Response to the COVID-19 Pandemic

The key public health function addressed by the highest number of digital innovations in the academic literature was communication and collaboration (264/561, 47.1% of digital innovations addressed this public health function; Table 4). Other key public health functions addressed by a large number of digital innovations were surveillance and monitoring (199/561, 35.5% of digital innovations), pandemic response (126/561, 22.5% of digital innovations), and screening and diagnostics (103/561, 18.4% of digital innovations).

**Table 4:** Number of digital innovations addressing each key public health function in the review of academic literature and nonacademic literature (review time frame for academic literature: January 1, 2020, to September 15, 2020; review time frame for nonacademic literature: January 1, 2020, to October 13, 2020)^a^.

Key public health function			Digital innovations, n (%)
**Review of academic literature (N=561)**			
	Communication and collaboration	264 (47.1)	
	Surveillance and monitoring	199 (35.5)	
	Pandemic response	126 (22.5)	
	Screening and diagnostics	103 (18.4)	
	Contact tracing	77 (13.7)	
	Forecasting	30 (5.3)	
	Signal or outbreak detection and validation	8 (1.4)	
**Review of nonacademic literature (N=497)**			
	Surveillance and monitoring	197 (39.6)	
	Pandemic response	169 (34.0)	
	Screening and diagnostics	167 (33.6)	
	Communication and collaboration	130 (26.2)	
	Contact tracing	52 (10.5)	
	Forecasting	27 (5.4)	
	Signal or outbreak detection and validation	6 (1.2)	

^a^For both the academic and nonacademic review, the number of digital innovations add up to more than the overall sample size (N) and the percentages add up to more than 100. This is because each digital innovation could be assigned more than one key public health function in our review.

The public health function addressed most commonly by digital innovations in the nonacademic literature was surveillance and monitoring (197/497, 39.6% of digital innovations), followed by pandemic response (169/497, 34% of digital innovations) and screening and diagnostics (167/497, 33.6% of digital innovations; Table 4). Compared with the academic literature, the function of communication and collaboration was addressed by a smaller number of innovations (130/497, 26.2% of digital innovations).

### Cross-analysis

For each country in which digital innovations were implemented, we cross-analyzed the number of high-level technology groups with which implemented innovations were associated. According to the academic literature, China had implemented digital innovations across the largest number of high-level technology groups (digital innovations were implemented with technologies from 15/17, 88% of the high-level technology groups). This was followed by the United States (14/17, 82% of high-level technology groups) and the United Kingdom (13/17, 76% of high-level technology groups). The EU or EEA countries with digital innovations covering the highest number of technology groups were France (11/17, 65% of high-level technology groups) and Italy (9/17, 53% of high-level technology groups). In the nonacademic literature review, the countries implementing technologies covering the largest number of high-level technology groups were the United States (15/17, 88% of high-level technology groups), the United Kingdom (12/17, 71% of high-level technology groups), China (10/17, 59% of high-level technology groups), and Italy (10/17, 59% of high-level technology groups). Tables presenting a further analysis of the implementation setting and high-level technology groups are presented in Multimedia Appendix 5.

For each key public health function, we cross-analyzed the high-level technology groups with which digital innovations were most associated. For communication and collaboration (the public health function addressed most frequently by digital innovations in the academic literature), most digital innovations incorporated technologies within the following high-level technology groups: *integrated and ubiquitous fixed and mobile networks* (88/561, 15.7% of digital innovations), *social media platforms* (56/561, 10% of digital innovations) *data analytics* *(including big data*) (42/561, 7.5% of digital innovations), and *web-based tools and platforms* (38/561, 6.8% of digital innovations). For surveillance and monitoring (the public health function addressed most frequently by digital innovations in the nonacademic literature), most digital innovations incorporated technologies within the following high-level technology groups: *integrated and ubiquitous fixed and mobile networks* (60/497, 12.1% of digital innovations), *web-based tools and platforms* (44/497, 8.9% of digital innovations), and *wearables (including ingestibles* (28/497, 5.6% of digital innovations). Tables presenting a further analysis of key public health functions and high-level technology groups are presented in Multimedia Appendix 5.

## Discussion

### Summary of Key Findings

Following the COVID-19 pandemic, actors worldwide turned to digital technologies to assist the public health response. This study suggests that the most common implementation settings for digital innovations implemented to tackle the COVID-19 pandemic were the United States, the United Kingdom, China, and India. Meanwhile, within the EU/EEA region, Italy, Spain, Germany, and France were the most common implementation settings. The study suggested that a high number of digital innovations implemented in response to the COVID-19 pandemic used technologies within the *integrated and ubiquitous fixed and mobile network* technology group, including cellular networks, smartphone and tablet computing devices, smartphone apps, and Bluetooth. Smartphone apps have been the specific technology most used by digital innovations in response to the COVID-19 pandemic, with uses ranging from contact-tracing apps [5,6,14-23] and self-assessment apps [24] to apps supporting population surveillance and monitoring of regulation compliance [25]. The study also found that *data analytics (including big data)* and *cognitive technologies*, the latter including AI and machine learning, have also been incorporated into many COVID-19–related digital innovations. According to the results of this study, communication and collaboration and surveillance and monitoring have been the public health functions most commonly addressed by digital innovations implemented in response to the COVID-19 pandemic. Both these functions have been addressed by a wide range of technologies, covering nearly all high-level technology groups used in this study. Other functions addressed by a large number of innovations were screening and diagnostics and pandemic response.

### Comparison With Other Studies

To the best of our knowledge, this study is the first to provide a broad overview of the geographical context in which digital technologies have been implemented in response to the COVID-19 pandemic. Mbunge et al [3] reviewed the evidence regarding leading countries in the application of AI models for COVID-19, finding that China was the country with the highest frequency in this respect, but did not review the geographic distribution of wider forms of digital innovation [3]. Although most digital innovations identified in our study were implemented in non-EU/EEA countries, it is also worth noting that in this study, EU/EEA countries featured more prominently as implementation settings when than an earlier scoping review that examined digital technologies implemented for infectious disease surveillance, prevention, and control more broadly [10].

This study’s findings on the types of digital technologies used by digital innovations have commonality with the results of other reviews on digital technologies and public health in response to the COVID-19 pandemic. In their review, for example, Vargo et al [4] found that the types of technological hardware most reported in relation to the health care sector were computerized tomography machines (in most cases discussed in combination with AI-based learning approaches) and mobile devices, with computers or mobile apps being among the most prominent forms of software used. Similarly, a review of digital technologies in health care conducted by Golinelli et al [2] found that many studies reported on the use of AI tools, big data analytics, mobile apps, and mobile tracing. More broadly, the wider array of digital technologies identified in this scoping review aligns with the digital technologies identified in other reviews. For example, in their review of digital technologies for COVID-19 prevention, surveillance, and containment, Mbunge et al [3] identified the following emerging technologies to be relevant in tackling COVID-19: AI, social media platforms, Internet of Medical Things, virtual or augmented reality, blockchain, additive manufacturing, 5G cellular technology and smart applications, geographic information systems, big data, and autonomous robots.

The findings of this study also illustrate certain differences from other reviews. For example, in the study by Vargo et al [4], video-based communication platforms were found to be a commonly reported on technological software. Meanwhile, Golinelli et al [2] reported a high number of articles reporting on telehealth or telemedicine. The difference between the high reportage of such technologies in other reviews and the relatively lower numbers found in this review may perhaps be explained by the fact that this study included only telemedicine-based innovations when the innovations had been implemented specifically to tackle the COVID-19 pandemic.

The study’s finding that digital technologies introduced in response to the COVID-19 pandemic are principally oriented toward 4 public health functions—communication and collaboration, surveillance and monitoring, screening and diagnostics, and pandemic response—is also in line with the findings of other reviews [1,2]. For example, Golinelli et al [2] identified the following 4 key *patient needs* that were addressed by technologies cited within the early scientific literature: *diagnosis*, *surveillance*, *prevention*, and *treatment*. Meanwhile, the review conducted by Budd et al [1] identified 4 overarching public health functions performed by technologies: *digital epidemiological surveillance*, *rapid case identification*, *interrupting community transmission*, and *public communication*. Each of these taxonomies broadly mirrors the functions most commonly addressed in this review, with the exception that, unlike the study by Golinelli et al [2], this study did not consider technologies related to COVID-19 treatment or therapeutics. In the sense that they emphasize the communicative or collaborative function of many COVID-19 digital innovations, the findings of this study are more closely aligned with the review conducted by Budd et al [1]. This emphasis on communication provides support for the broader literature on the role of social media in public health communication during the pandemic [26]. The literature has highlighted the role of social media in facilitating forms of communication such as scientific exchange and the transmission of information from formal public health agencies and other bodies, as well as the potential for such platforms to act as vectors for the spread of misinformation [1,26].

In conducting this scoping review, we encountered evidence of a range of barriers to the successful implementation of digital innovation, including potential risks. In addition to the limitations of the technologies themselves, these include investment and financial barriers, infrastructural barriers (including a lack of required physical and network infrastructure), human resource barriers, data availability and quality barriers, social barriers (including low uptake and low access to technologies), ethical barriers (including privacy concerns and risks of increased socioeconomic inequality), security and safety barriers, and legal or regulatory barriers. In their review, Budd et al [1] identified similar legal, ethical, and privacy barriers, as well as organizational and workforce barriers, to the implementation of technologies for the COVID-19 pandemic. The extent to which these factors present an obstacle to the implementation of technologies depends on the specific contexts (eg, geographical, cultural, political, and economic) within which technologies are developed and implemented. Therefore, the literature suggests that an effective rollout of technologies will require interventions tailored to the specific characteristics of target regions, recognizing both barriers and enablers that may exist [27]. For instance, in regions without the necessary infrastructure to support cellular and data coverage, automated applications that do not require continuous network access may be more appropriate than other applications [27].

Although this study presents evidence regarding the technologies used by digital innovations, the public health functions addressed, and barriers to implementation, it has not systematically examined the performance of individual technologies or the extent to which technologies have been evaluated or comparatively assessed (see the *Limitations* section). The wider literature provides examples of evaluative studies in specific contexts, including statistical evaluations of diagnostic accuracy [28], epidemic modeling [29,30], and qualitative evaluations [31], which demonstrate that the performance of digital technologies implemented in response to the COVID-19 pandemic can vary significantly, depending not only on endogenous technological factors but also on broader exogenous factors, including legal, infrastructural, and social issues [28,29,30,31]. It is also evident that a large number of the technologies introduced in response to the COVID-19 pandemic have not yet been formally evaluated or assessed.

Therefore, the rapid proliferation of digital public health technologies in response to the COVID-19 pandemic has underscored the need for further studies to evaluate the performance of emerging digital technologies, as well as rigorous oversight mechanisms [1,3]. Such approaches should help not only to verify the performance of new technologies but also to identify the underpinning barriers that stand in the way of those technologies realizing their potential. At the same time, oversight mechanisms should also help to strike a balance between the opportunities presented by new innovations and potential risks, such as ethical and privacy risks, that they may pose [32].

### Limitations

This study has sought to provide a broad characterization of the evidence regarding the implementation of digital innovations to tackle the COVID-19 pandemic. This study followed a systematic approach in line with the PRISMA checklist for scoping reviews. Drawing on 2 scoping reviews—a review of academic literature, supported by a supplementary, experimental review of nonacademic literature—the review considers evidence from a wide range of sources, from peer-reviewed publications to news articles, press releases, and blogs. Although the methodological approach is well suited to the objectives of the study, it is also subject to several limitations.

The first limitation of the study relates to the scope of information sources. For the review of academic literature, we relied on 2 databases, EMBASE and Scopus, supplemented by structured targeted searches using Google Scholar. Similarly, our review of nonacademic literature was also limited in that it only considered articles published by a selected set of information sources. For example, in focusing on information sources available within Feedly, the nonacademic search strategy did not include national public health institute websites (although our search strategy identified several digital innovations developed and implemented by public health institutes). We cannot rule out the possibility that running the searches in additional databases, including those used by other reviews of digital technology use for the COVID-19 pandemic, might have led us to identify further examples of digital innovations implemented in response to the COVID-19 pandemic.

We adopted broad inclusion criteria during study selection to maximize the scope of the included evidence. However, it was also necessary to limit the review’s scope to keep it manageable within the resources available for this study. Our decision to focus on *implemented* digital innovations, thereby excluding innovations still at the conceptual stage, was an example of this. Another was our focus on specific key public health functions, meaning that innovations oriented toward other functions were excluded. In both cases, such decisions led to the inevitable exclusion of digital innovations developed in response to the COVID-19 pandemic.

Another limitation of this scoping review was related to the categories used to code technologies and key public health functions. To extract data, we used drop-down menus to classify digital innovations by technology (specific digital technology and high-level technology groups) and by key public health function. The categories used for the drop-down menus (described earlier as key study variables) were carefully selected after several discussions between team members and drew on earlier studies [10]. Although these categories helped classify and organize the data for the purposes of quantitative analysis, inevitably, there is also an element of subjectivity in the application of these categories to digital innovations. It is also not claimed that these categories are definitive or exhaustive in any way. They represent only one approach to classifying implemented technologies and the role they have performed in supporting public health efforts.

We also faced some technical limitations in analyzing data on the types of nonacademic sources included in the review. Initially, the sources were categorized as news articles, blog posts, or press releases. However, as most sources were news articles, it was decided to merge these 3 categories into one during the analysis stage. The decision also reflected the challenges faced during the export of the included articles into a Microsoft Excel file (a measure taken to mitigate the potential risk of URL changes). With the article content exported to Microsoft Excel, it was not always possible to determine the original format (eg, whether a source was an original news article or an article based on a press release).

Finally, while reviewing evidence on the technologies used by digital innovations, the public health functions addressed, and the key barriers to implementation, a systematic evaluation of the performance of individual technologies and innovations is beyond the scope of this study. The incorporation of an evaluative aspect into the study was not feasible because of the limited amount of information on the performance of digital technologies within the reviewed sources, including the lack of evidence of formal evaluation or assessments undertaken. This study highlights the need for further evaluative studies and oversight mechanisms moving forward.

### Conclusions

In this study, scoping reviews of academic and nonacademic sources were used to obtain an overview of the evidence regarding implemented digital innovations to tackle the COVID-19 pandemic. This scoping review sought to gain an understanding of the characteristics of the literature reporting on digital technology use for COVID-19 and an understanding of the number, nature, and geographical distribution of digital innovations implemented during the first 10 months of the COVID-19 pandemic. This study built on the evidence base established by existing reviews by incorporating new sources and approaches to analysis. This study highlighted key trends related to the implementation settings, technologies used, and public health functions addressed by COVID-19–related digital innovations. This study also identified a wide-ranging set of barriers and risks that may affect the effective implementation of digital technologies for the COVID-19 pandemic. The existence of such barriers highlights the need for contextually appropriate technological interventions. Although this study did not critically evaluate the effectiveness of digital innovations, the findings from the broader literature point to the fact that technologies introduced as part of the COVID-19 pandemic response demonstrate varying levels of performance and that, in many cases, technologies have yet to be evaluated or comparatively assessed. These findings highlight the need for further evaluation and oversight mechanisms to balance opportunities and risks.

## Appendix

Multimedia Appendix 1 PRISMA-ScR (Preferred Reporting Items for Systematic Reviews and Meta-Analyses extension for Scoping Reviews) checklist.

Multimedia Appendix 2 Search terms.

Multimedia Appendix 3 Inclusion and exclusion criteria.

Multimedia Appendix 4 Extraction templates.

Multimedia Appendix 5 Cross-analysis tables.

## Abbreviations

AI: artificial intelligence
EU/EEA: European Union/European Economic Area
PRISMA: Preferred Reporting Items for Systematic Reviews and Meta-Analyses
PRISMA-ScR: Preferred Reporting Items for Systematic Reviews and Meta-Analyses extension for Scoping Reviews
