# Supplementary material for: Assessing the Implementation of Digital Innovations in Response to the COVID-19 Pandemic to Address Key Public Health Functions: Scoping Review of Academic and Nonacademic Literature
Source: JMIR Public Health Surveill. 2022 Jul 6;8(7):e34605. doi: 10.2196/34605 (PMC9301563; doi:10.2196/34605)
Supplement: Multimedia Appendix 2 [file publichealth_v8i7e34605_app2.docx]

# Appendix 2: Search terms

Table A2-1: Search terms used for the review of academic literature: Embase: Title, abstract, keywords and Emtree headings (review time frame: January 1, 2020, to September 15, 2020).

| Search number | Search terms |
| --- | --- |
| #1 | ‘2019-nCov':ab,ti,kw OR 'COVID-19':ab,ti,kw OR ‘COVID19’:ab,ti,kw OR 'SARS-CoV-2':ab,ti,kw OR ‘severe acute respiratory syndrome coronavirus 2’:ab,ti,kw OR ‘SARS-COV2’:ab,ti,kw OR ‘nCOV-19’:ab,ti,kw OR 'coronavirus disease 2019'/exp |
| #2 | 'screen*':ab,ti,kw OR 'diagnos*':ab,ti,kw OR 'diagnosis'/exp OR 'surveillance':ab,ti,kw OR 'monitor*':ab,ti,kw OR 'predict*':ab,ti,kw OR 'forecast*':ab,ti,kw OR 'forecasting'/exp OR 'detect*':ab,ti,kw OR 'validat*':ab,ti,kw OR 'respon*':ab,ti,kw OR 'communicat*':ab,ti,kw OR 'collaborat*':ab,ti,kw OR 'epidemiolog*':ab,ti,kw OR 'epidemiology'/exp OR 'ipc':ab,ti,kw OR 'prevent*':ab,ti,kw OR 'infection prevention'/exp OR 'control*':ab,ti,kw OR 'infection control'/exp OR 'test*':ab,ti,kw OR 'track*':ab,ti,kw OR 'trace*':ab,ti,kw OR 'tracing':ab,ti,kw OR 'contact examination'/exp OR 'notif*':ab,ti,kw OR 'disease notification'/exp |
| #3 | ‘digital technolog*’:ab,ti,kw OR ‘information technolog*’:ab,ti,kw OR 'information technology'/exp OR ‘communication technolog*’:ab,ti,kw OR ‘communications technolog*’:ab,ti,kw OR ‘ict’:ab,ti,kw OR ‘new technolog*’:ab,ti,kw OR ‘digital innovation*’:ab,ti,kw OR ‘digital technolog*’:ab,ti,kw OR ‘emerging technolog*':ab,ti,kw OR ‘disruptive technolog*’:ab,ti,kw OR ‘3d print*’:ab,ti,kw OR ‘printing, three-dimensional’:ab,ti,kw OR 'three dimensional printing'/exp OR ‘additive manufacturing’:ab,ti,kw OR ‘automation’:ab,ti,kw OR ‘drone’:ab,ti,kw OR ‘UAV’:ab,ti,kw OR ‘unmanned aerial vehicle*’:ab,ti,kw OR ‘autonomous vehicle*’:ab,ti,kw OR ‘automation’:ab,ti,kw OR ‘robotic*’:ab,ti,kw OR 'robotics'/exp OR ‘blockchain’:ab,ti,kw OR 'distributed ledger technolog*':ab,ti,kw OR ‘cloud’:ab,ti,kw OR ‘cloud computing’:ab,ti,kw OR 'cloud computing'/exp OR ‘artificial intelligence’:ab,ti,kw OR 'artificial intelligence'/exp OR 'expert system*':ab,ti,kw OR ‘machine learning’:ab,ti,kw OR 'machine learning'/exp OR ‘deep learning’:ab,ti,kw OR 'natural language processing':ab,ti,kw OR 'neural network*':ab,ti,kw OR 'artificial neural network'/exp OR ‘crowdsourcing’:ab,ti,kw OR ‘crowd sourcing’:ab,ti,kw OR 'crowdsourcing'/exp OR ‘big data’:ab,ti,kw OR 'big data'/exp OR ‘data mining’:ab,ti,kw OR 'data mining'/exp OR ‘datamining’:ab,ti,kw OR ‘social media’:ab,ti,kw OR 'social media'/exp OR ‘facebook’:ab,ti,kw OR ‘instagram’:ab,ti,kw OR ‘twitter’:ab,ti,kw OR 'health informatics':ab,ti,kw OR 'parallel computing':ab,ti,kw OR ‘digital health’:ab,ti,kw OR 'eHealth':ab,ti,kw OR 'mHealth':ab,ti,kw OR 'EHR':ab,ti,kw OR 'EMR':ab,ti,kw OR 'electronic health record*':ab,ti,kw OR 'electronic medical record*':ab,ti,kw OR ‘satellite imagery’:ab,ti,kw OR ‘satellite imaging’:ab,ti,kw OR ‘satellite communication* ‘:ab,ti,kw OR ‘satellite communications’:ab,ti,kw OR ‘remote sensing’:ab,ti,kw OR ‘augmented reality’:ab,ti,kw OR ‘virtual reality’:ab,ti,kw OR ‘virtual setting*’:ab,ti,kw OR 'virtual reality'/exp OR ((‘cellular phone*’:ab,ti,kw OR ‘cell phone*’:ab,ti,kw OR ‘mobile phone*':ab,ti,kw) AND (‘health technol*’:ab,ti,kw OR ‘biomedical technol*’:ab,ti,kw OR ‘medical technol*’:ab,ti,kw)) OR ‘3G’:ab,ti,kw OR ‘4G’:ab,ti,kw OR ‘5G’:ab,ti,kw OR ‘internet of things’:ab,ti,kw OR ‘iot’:ab,ti,kw OR 'internet of things'/exp OR 'wireless sensor network*':ab,ti,kw OR 'biosensor*':ab,ti,kw OR 'lab-on-chip':ab,ti,kw OR ‘nanotech*’:ab,ti,kw OR ‘smart fabric*’:ab,ti,kw OR ‘wearables’:ab,ti,kw OR ‘wearable technol*’:ab,ti,kw OR ‘ingestibles’:ab,ti,kw OR (‘ingestible’:ab,ti,kw AND (‘technol*’:ab,ti,kw OR ‘sensor*’:ab,ti,kw)) OR ‘wearable electronic device*’:ab,ti,kw OR ‘quantum comput*’:ab,ti,kw |
| #4 | **#1 AND #2 AND #3** |

Table A2-2: Summary of search results from Embase search (review time frame: January 1, 2020, to September 15, 2020).

| **Database** | **Total number of results** | **Total number of results minus duplicates from other database searches** |
| --- | --- | --- |
| Embase | 2613 | 2613 |

Table A2-3: Search terms used for the review of academic literature: Scopus: Title, abstract and keywords with proximity functions (review time frame: January 1, 2020, to September 15, 2020).

| Search number | Search terms |
| --- | --- |
| #1 | TITLE-ABS-KEY ("2019-nCoV" OR "COVID-19" OR “COVID19” OR “SARS-CoV-2” OR “severe acute respiratory syndrome coronavirus 2” OR “SARS-COV2” OR “nCOV-19”) |
| #2 | TITLE-ABS-KEY (screen* OR diagnos* OR surveillance OR monitor* OR predict* OR forecast* OR detect* OR validat* OR respon* OR communicat* OR collaborat* OR epidemiolog* OR “IPC” OR prevent* OR control* OR test* OR track* OR trace* OR tracing OR notif*) |
| #3 | TITLE-ABS-KEY ( (digital W/2 technolog*) OR (information W/2 technolog*) OR (communication W/2 technolog*) OR ICT OR ("new technolog*") OR (digital W/2 innovation*) OR (emerging W/2 technolog*) OR (disruptive W/2 technolog*) OR (3D W/2 print*) OR (three-dimensional W/2 print*) OR "additive manufacturing" OR drone* OR UAV OR "unmanned aerial vehicle*" OR "autonomous vehicle*" OR automation OR robotic* OR blockchain OR "distributed ledger technolog*" OR cloud OR "artificial intelligence" OR "expert system*" OR "machine learning" OR "deep learning" OR "natural language processing" OR "neural network*" OR crowdsourcing OR "crowd sourcing" OR "big data" OR (data W/2 mining) OR (data W/2 mine) OR datamining OR "social media" OR facebook OR instagram OR twitter OR "digital health" OR eHealth OR mHealth OR EHR OR EMR OR "electronic health record*" OR "electronic medical record*" OR (satellite W/2 imag*) OR (satellite W/2 communication*) OR "remote sensing technol*" OR (virtual W/2 reality) OR (virtual W/2 setting) OR (augmented W/2 reality) OR (((cell* w/0 phone*) OR (cell* w/0 telephone*) OR ("mobile phone*")) AND ((health W/2 technol*) OR (biomedical W/2 technol*) OR (medical W/2 technol*))) OR 3G OR 4G OR 5G OR "internet of things" OR "iot" OR "wireless sensor network*" OR biosensor* OR lab-on-chip OR nanotech* OR "smart fabric" OR wearables OR "wearable technol*" OR ingestibles OR (ingestible* AND (technolog* OR sensor*)) OR "quantum comput*" ) |
| #4 | **#1 AND #2 AND #3** |

Table A2-4: Summary of search results from Scopus search (review time frame: January 1, 2020, to September 15, 2020).

| **Database** | **Total number of results** | **Total number of results minus duplicates from other database searches** |
| --- | --- | --- |
| Scopus | 2405 | 997 |

Table A2-5: Search terms used for the review of nonacademic literature – Feedly search (review time frame: 1st January 2020 to 13th October 2020).

| Search number | Search terms |
| --- | --- |
| **#1** | ("2019-nCoV" OR "covid-19" OR "SARS-CoV-2" OR "coronavirus" OR "corona virus") |
| **#2** | (screen OR diagnose OR surveillance OR monitor OR predict OR forecast OR detect OR validate OR respond OR communicate OR collaborate OR epidemiology OR ipc OR prevent OR control OR test OR track OR trace OR notify) |
| **#3** | ("digital technology" OR "information technology" OR "communication technology" OR "communications technology" OR "ICT" OR "new technology" OR "digital innovation" OR "emerging technology" OR "disruptive technology" OR "3D printing" OR "three-dimensional printing" OR “additive manufacturing" OR "drone" OR "drones" OR "UAV" OR "unmanned aerial vehicle" OR "autonomous vehicle" OR "automation" OR "robotic" OR "robotics" OR "blockchain" OR "distributed ledger technology" OR "cloud" OR "cloud computing" OR "artificial intelligence" OR “AI” OR "expert system" OR "machine learning" OR "deep learning" OR "natural language processing" OR "neural network" OR “neural networks" OR “crowdsourcing" OR "crowd sourcing" OR "big data" OR "data mining" OR "datamining" OR "social media" OR "facebook" OR "instagram" OR "twitter" OR "health informatics" OR "parallel computing" OR "digital health" OR "eHealth" OR "mHealth" OR "EHR" OR "EMR" OR "electronic health records" OR "electronic medical records" OR "GIS" OR "image processing" OR "satellite imagery" OR "satellite imaging" OR "satellite communication" OR "remote sensing" OR "augmented reality" OR "virtual reality" OR "virtual setting" OR "3G" OR "4G" OR "5G" OR "internet of things" OR "IoT" OR "wireless sensor network" OR "biosensor" OR "lab-on-chip" OR "nanotechnology" OR "smart fabric" OR "wearables" OR "wearable technology" OR "wearable electronic device" OR "ingestibles" OR "ingestible technology" OR "sensor" OR "quantum computing" OR "health technology" OR "biomedical technology" OR "medical technology") |
| **#4** | **#1 AND #2 AND #3** |

Table A2-6: Summary of search results from Feedly search (review time frame: January 1, 2020, to October 13, 2020).

| **Database** | **Total number of results** | **Total number of results minus duplicates** |
| --- | --- | --- |
| Feedly | 4537 | 4393 |
