# Supplementary material for: Assessing the Implementation of Digital Innovations in Response to the COVID-19 Pandemic to Address Key Public Health Functions: Scoping Review of Academic and Nonacademic Literature
Source: JMIR Public Health Surveill. 2022 Jul 6;8(7):e34605. doi: 10.2196/34605 (PMC9301563; doi:10.2196/34605)
Supplement: Multimedia Appendix 3 [file publichealth_v8i7e34605_app3.doc]

# Appendix 3: Inclusion and exclusion criteria

Table A3-1. Inclusion and exclusion criteria used for the academic literature search (review time frame: January 1, 2020, to September 15, 2020).

|  | **Inclusion** | **Exclusion** |
| --- | --- | --- |
| **Population/Topic of interest** | COVID-19 surveillance, prevention and control in humans | COVID-19 surveillance, prevention and control in plants and animals |
| **Intervention** | Any digital technology implemented in a real-world setting to tackle COVID-19 | Digital technologies not implemented in a real-world setting to tackle COVID-19 (e.g. conceptual work, prototypes and digital technologies that are being tested (for example, to show technical feasibility) but have not reached the stage of being used for COVID-19 surveillance, prevention and control) |
| **Comparison** | Any or no comparison | N/A |
| **Outcome** | Any benefit or disruption to COVID-19 public health key functions | N/A |
| **Study type** | Completed research studies, research protocols, conference proceedings with full text, theoretical papers, commentaries, letters, working papers, books and book chapters | Conference proceedings that do not include full text |
| **Date** | Published from January 1, 2020, to date of search (September 15, 2020) | Published before January 2020 |
| **Language** | English articles included and extracted  Non-English articles included in study selection, but data only extracted from English abstracts or summaries if available (no data extracted from non-English language text) | No exclusion based on language, but data only extracted from abstracts and articles available in English |

Table A3-2. Inclusion and exclusion criteria for the nonacademic literature search (using the news aggregation software, Feedly) (review time frame: January 1, 2020, to October 13, 2020).

|  | **Inclusion** | **Exclusion** |
| --- | --- | --- |
| **Population/Topic of interest** | COVID-19 surveillance, prevention and control in humans | COVID-19 surveillance, prevention and control in plants and animals |
| **Intervention** | Any digital technology implemented in a real-world setting to tackle COVID-19 | Digital technologies not implemented in a real-world setting to tackle COVID-19 (e.g. conceptual work, prototypes and digital technologies that are being tested (for example, to show technical feasibility) but have not reached the stage of being used for COVID-19 surveillance, prevention and control) |
| **Comparison** | Any or no comparison | N/A |
| **Outcome** | Any potential benefit or disruption to public health key functions | N/A |
| **Study type** | Any news article, press release, blogpost, or other news story captured by Feedly search  Any news article, press release, blog post, or other news story identified in the targeted search and that was not already included in the Feedly search | N/A |
| **Date** | Published from January 1, 2020, to four weeks after date of search (October 13, 2020) | Published before January 2020 |
| **Language** | English articles included and extracted | Non-English articles |

Source: RAND Europe
