# Supplementary material for: Assessing the Implementation of Digital Innovations in Response to the COVID-19 Pandemic to Address Key Public Health Functions: Scoping Review of Academic and Nonacademic Literature
Source: JMIR Public Health Surveill. 2022 Jul 6;8(7):e34605. doi: 10.2196/34605 (PMC9301563; doi:10.2196/34605)
Supplement: Multimedia Appendix 4 [file publichealth_v8i7e34605_app4.docx]

# Appendix 4: Extraction templates

Table A4-1. Extraction template and drop-down menus for the review of academic literature (review time frame: January 1, 2020, to September 15, 2020).

| Article type | Study type | Is it a comparative study? | Geographical context of technology implementation (EU/EEA, Non-EU/EEA or Both) | Specific country context of technology implementation | Contributing Region (EU/EEA, Non-EU/EEA or Both)  (First, last and corresponding author) | Key contributing countries  (First, last and corresponding author) |
| --- | --- | --- | --- | --- | --- | --- |
| Research article  Brief journal comment/editorial/letter/opinion  Detailed journal perspective/policy review/practice review  Working paper  Book  Book chapter  Conference proceeding  Magazine article  News article  Press release  Blog post | Mathematical model/simulation  Primary study  Scoping review  Systematic review  Other literature review  NA  Unclear | Yes  No  NA | EU/EEA | Global  UN Agency  European Union  North America  South America  Sub-Saharan Africa  Afghanistan  Albania  Algeria  Andorra  Angola  Antigua and Barbuda  Argentina  Armenia  Australia  Austria  Azerbaijan  Bahamas  Bahrain  Bangladesh  Barbados  Belarus  Belgium  Belize  Benin  Bhutan  Bolivia  Bosnia and Herzegovina  Botswana  Brazil  Brunei  Bulgaria  Burkina Faso  Burundi  Côte d'Ivoire  Cabo Verde  Cambodia  Cameroon  Canada  Central African Republic  Chad  Chile  China (mainland)  Colombia  Comoros  Congo (Congo-Brazzaville)  Costa Rica  Croatia  Cuba  Cyprus  Czechia (Czech Republic)  Democratic Republic of the Congo  Denmark  Djibouti  Dominica  Dominican Republic  Ecuador  Egypt  El Salvador  Equatorial Guinea  Eritrea  Estonia  Eswatini  Ethiopia  Fiji  Finland  France  Gabon  Gambia  Georgia  Germany  Ghana  Greece  Grenada  Guatemala  Guinea  Guinea-Bissau  Guyana  Haiti  Holy See  Honduras  Hong Kong  Hungary  Iceland  India  Indonesia  Iran  Iraq  Ireland  Israel  Italy  Jamaica  Japan  Jordan  Kazakhstan  Kenya  Kiribati  Kuwait  Kyrgyzstan  Laos  Latvia  Lebanon  Lesotho  Liberia  Libya  Liechtenstein  Lithuania  Luxembourg  Madagascar  Malawi  Malaysia  Maldives  Mali  Malta  Marshall Islands  Mauritania  Mauritius  Mexico  Micronesia  Moldova  Monaco  Mongolia  Montenegro  Morocco  Mozambique  Myanmar  Namibia  Nauru  Nepal  Netherlands  New Zealand  Nicaragua  Niger  Nigeria  North Korea  North Macedonia  Norway  Oman  Pakistan  Palau  Palestine State  Panama  Papua New Guinea  Paraguay  Peru  Philippines  Poland  Portugal  Qatar  Romania  Russia  Rwanda  Saint Kitts and Nevis  Saint Lucia  Saint Vincent and the Grenadines  Samoa  San Marino  Sao Tome and Principe  Saudi Arabia  Senegal  Serbia  Seychelles  Sierra Leone  Singapore  Slovakia  Slovenia  Solomon Islands  Somalia  South Africa  South Korea  South Sudan  Spain  Sri Lanka  Sudan  Suriname  Sweden  Switzerland  Syria  Taiwan  Tajikistan  Tanzania  Thailand  Timor-Leste  Togo  Tonga  Trinidad and Tobago  Tunisia  Turkey  Turkmenistan  Tuvalu  Uganda  Ukraine  United Arab Emirates  United Kingdom  United States of America  Uruguay  Uzbekistan  Vanuatu  Venezuela  Vietnam  Yemen  Zambia  Zimbabwe  MNC  Not reported  NA | EU/EEA | Afghanistan  Albania  Algeria  Andorra  Angola  Antigua and Barbuda  Argentina |
|  |  |  | Non-EU/EEA |  | Non-EU/EEA |  |
|  |  |  | Both |  | Both |  |
|  |  |  | Not reported |  | Not reported |  |
|  |  |  | NA |  |  |  |
|  |  |  |  |  |  |  |
|  |  |  |  |  |  |  |
|  |  |  |  |  |  | Armenia |
|  |  |  |  |  |  | Australia |
|  |  |  |  |  |  | Austria |
|  |  |  |  |  |  | Azerbaijan |
|  |  |  |  |  |  | Bahamas |
|  |  |  |  |  |  | Bahrain |
|  |  |  |  |  |  | Bangladesh |
|  |  |  |  |  |  | Barbados |
|  |  |  |  |  |  | Belarus |
|  |  |  |  |  |  | Belgium |
|  |  |  |  |  |  | Belize |
|  |  |  |  |  |  | Benin |
|  |  |  |  |  |  | Bhutan |
|  |  |  |  |  |  | Bolivia |
|  |  |  |  |  |  | Bosnia and Herzegovina |
|  |  |  |  |  |  | Botswana |
|  |  |  |  |  |  | Brazil |
|  |  |  |  |  |  | Brunei |
|  |  |  |  |  |  | Bulgaria |
|  |  |  |  |  |  | Burkina Faso |
|  |  |  |  |  |  | Burundi |
|  |  |  |  |  |  | Côte d'Ivoire |
|  |  |  |  |  |  | Cabo Verde |
|  |  |  |  |  |  | Cambodia |
|  |  |  |  |  |  | Cameroon |
|  |  |  |  |  |  | Canada |
|  |  |  |  |  |  | Central African Republic |
|  |  |  |  |  |  | Chad |
|  |  |  |  |  |  | Chile |
|  |  |  |  |  |  | China (mainland) |
|  |  |  |  |  |  | Colombia |
|  |  |  |  |  |  | Comoros |
|  |  |  |  |  |  | Congo (Congo-Brazzaville) |
|  |  |  |  |  |  | Costa Rica |
|  |  |  |  |  |  | Croatia |
|  |  |  |  |  |  | Cuba |
|  |  |  |  |  |  | Cyprus |
|  |  |  |  |  |  | Czechia (Czech Republic) |
|  |  |  |  |  |  | Democratic Republic of the Congo |
|  |  |  |  |  |  | Denmark |
|  |  |  |  |  |  | Djibouti |
|  |  |  |  |  |  | Dominica |
|  |  |  |  |  |  | Dominican Republic |
|  |  |  |  |  |  | Ecuador |
|  |  |  |  |  |  | Egypt |
|  |  |  |  |  |  | El Salvador |
|  |  |  |  |  |  | Equatorial Guinea |
|  |  |  |  |  |  | Eritrea |
|  |  |  |  |  |  | Estonia |
|  |  |  |  |  |  | Eswatini |
|  |  |  |  |  |  | Ethiopia |
|  |  |  |  |  |  | Fiji |
|  |  |  |  |  |  | Finland |
|  |  |  |  |  |  | France |
|  |  |  |  |  |  | Gabon |
|  |  |  |  |  |  | Gambia |
|  |  |  |  |  |  | Georgia |
|  |  |  |  |  |  | Germany |
|  |  |  |  |  |  | Ghana |
|  |  |  |  |  |  | Greece |
|  |  |  |  |  |  | Grenada |
|  |  |  |  |  |  | Guatemala |
|  |  |  |  |  |  | Guinea |
|  |  |  |  |  |  | Guinea-Bissau |
|  |  |  |  |  |  | Guyana |
|  |  |  |  |  |  | Haiti |
|  |  |  |  |  |  | Holy See |
|  |  |  |  |  |  | Honduras |
|  |  |  |  |  |  | Hong Kong |
|  |  |  |  |  |  | Hungary |
|  |  |  |  |  |  | Iceland |
|  |  |  |  |  |  | India |
|  |  |  |  |  |  | Indonesia |
|  |  |  |  |  |  | Iran |
|  |  |  |  |  |  | Iraq |
|  |  |  |  |  |  | Ireland |
|  |  |  |  |  |  | Israel |
|  |  |  |  |  |  | Italy |
|  |  |  |  |  |  | Jamaica |
|  |  |  |  |  |  | Japan |
|  |  |  |  |  |  | Jordan |
|  |  |  |  |  |  | Kazakhstan |
|  |  |  |  |  |  | Kenya |
|  |  |  |  |  |  | Kiribati |
|  |  |  |  |  |  | Kuwait |
|  |  |  |  |  |  | Kyrgyzstan |
|  |  |  |  |  |  | Laos |
|  |  |  |  |  |  | Latvia |
|  |  |  |  |  |  | Lebanon |
|  |  |  |  |  |  | Lesotho |
|  |  |  |  |  |  | Liberia |
|  |  |  |  |  |  | Libya |
|  |  |  |  |  |  | Liechtenstein |
|  |  |  |  |  |  | Lithuania |
|  |  |  |  |  |  | Luxembourg |
|  |  |  |  |  |  | Madagascar |
|  |  |  |  |  |  | Malawi |
|  |  |  |  |  |  | Malaysia |
|  |  |  |  |  |  | Maldives |
|  |  |  |  |  |  | Mali |
|  |  |  |  |  |  | Malta |
|  |  |  |  |  |  | Marshall Islands |
|  |  |  |  |  |  | Mauritania |
|  |  |  |  |  |  | Mauritius |
|  |  |  |  |  |  | Mexico |
|  |  |  |  |  |  | Micronesia |
|  |  |  |  |  |  | Moldova |
|  |  |  |  |  |  | Monaco |
|  |  |  |  |  |  | Mongolia |
|  |  |  |  |  |  | Montenegro |
|  |  |  |  |  |  | Morocco |
|  |  |  |  |  |  | Mozambique |
|  |  |  |  |  |  | Myanmar |
|  |  |  |  |  |  | Namibia |
|  |  |  |  |  |  | Nauru |
|  |  |  |  |  |  | Nepal |
|  |  |  |  |  |  | Netherlands |
|  |  |  |  |  |  | New Zealand |
|  |  |  |  |  |  | Nicaragua |
|  |  |  |  |  |  | Niger |
|  |  |  |  |  |  | Nigeria |
|  |  |  |  |  |  | North Korea |
|  |  |  |  |  |  | North Macedonia |
|  |  |  |  |  |  | Norway |
|  |  |  |  |  |  | Oman |
|  |  |  |  |  |  | Pakistan |
|  |  |  |  |  |  | Palau |
|  |  |  |  |  |  | Palestine State |
|  |  |  |  |  |  | Panama |
|  |  |  |  |  |  | Papua New Guinea |
|  |  |  |  |  |  | Paraguay |
|  |  |  |  |  |  | Peru |
|  |  |  |  |  |  | Philippines |
|  |  |  |  |  |  | Poland |
|  |  |  |  |  |  | Portugal |
|  |  |  |  |  |  | Qatar |
|  |  |  |  |  |  | Romania |
|  |  |  |  |  |  | Russia |
|  |  |  |  |  |  | Rwanda |
|  |  |  |  |  |  | Saint Kitts and Nevis |
|  |  |  |  |  |  | Saint Lucia |
|  |  |  |  |  |  | Saint Vincent and the Grenadines |
|  |  |  |  |  |  | Samoa |
|  |  |  |  |  |  | San Marino |
|  |  |  |  |  |  | Sao Tome and Principe |
|  |  |  |  |  |  | Saudi Arabia |
|  |  |  |  |  |  | Senegal |
|  |  |  |  |  |  | Serbia |
|  |  |  |  |  |  | Seychelles |
|  |  |  |  |  |  | Sierra Leone |
|  |  |  |  |  |  | Singapore |
|  |  |  |  |  |  | Slovakia |
|  |  |  |  |  |  | Slovenia |
|  |  |  |  |  |  | Solomon Islands |
|  |  |  |  |  |  | Somalia |
|  |  |  |  |  |  | South Africa |
|  |  |  |  |  |  | South Korea |
|  |  |  |  |  |  | South Sudan |
|  |  |  |  |  |  | Spain |
|  |  |  |  |  |  | Sri Lanka |
|  |  |  |  |  |  | Sudan |
|  |  |  |  |  |  | Suriname |
|  |  |  |  |  |  | Sweden |
|  |  |  |  |  |  | Switzerland |
|  |  |  |  |  |  | Syria |
|  |  |  |  |  |  | Taiwan |
|  |  |  |  |  |  | Tajikistan |
|  |  |  |  |  |  | Tanzania |
|  |  |  |  |  |  | Thailand |
|  |  |  |  |  |  | Timor-Leste |
|  |  |  |  |  |  | Togo |
|  |  |  |  |  |  | Tonga |
|  |  |  |  |  |  | Trinidad and Tobago |
|  |  |  |  |  |  | Tunisia |
|  |  |  |  |  |  | Turkey |
|  |  |  |  |  |  | Turkmenistan |
|  |  |  |  |  |  | Tuvalu |
|  |  |  |  |  |  | Uganda |
|  |  |  |  |  |  | Ukraine |
|  |  |  |  |  |  | United Arab Emirates |
|  |  |  |  |  |  | United Kingdom |
|  |  |  |  |  |  | United States of America |
|  |  |  |  |  |  | Uruguay |
|  |  |  |  |  |  | Uzbekistan |
|  |  |  |  |  |  | Vanuatu |
|  |  |  |  |  |  | Venezuela |
|  |  |  |  |  |  | Vietnam |
|  |  |  |  |  |  | Yemen |
|  |  |  |  |  |  | Zambia |
|  |  |  |  |  |  | Zimbabwe |
|  |  |  |  |  |  | Not reported |
|  |  |  |  |  |  | NA |
|  |  |  |  |  |  |  |
|  |  |  |  |  |  |  |
|  |  |  |  |  |  |  |
|  |  |  |  |  |  |  |
|  |  |  |  |  |  |  |
|  |  |  |  |  |  |  |
|  |  |  |  |  |  |  |

| High-level technology group 1 | | Specific digital technology 1 | High-level technology group 2 | | Specific digital technology 2 | |
| --- | --- | --- | --- | --- | --- | --- |
| Advanced manufacturing technologies  Autonomous devices and systems  Blockchain / Distributed ledger technology  Cloud computing / cloud-based networks  Cognitive technologies  Crowdsourcing platforms  Data analytics (including big data)  eHealth  Imaging and sensing technologies (including GIS)  Immersive technologies  Integrated and ubiquitous fixed and mobile networks  Internet of things (IoT)  Nanotechnology and microsystems  Online tools and platforms  Quantum computing  Simulation  Social media platforms  Wearables (including ingestibles) | 3D-printing  Drones  Robotics  Blockchain / Distributed ledger technology  Cloud computing / cloud-based networks  Artificial intelligence (AI)  Expert systems  Facial recognition  Machine learning (ML)  Natural language processing  (Artificial) neural networks  Crowdsourcing  Data mining  Data analytics  Big data  Bioinformatics  Parallel computing  Social media and mobile data analysis  Digital health /eHealth / mHealth  Electronic health records (EHRs)  Telemedicine  Geographic information systems (GIS)  Health informatics  Image processing  Infrared sensing  Satellite communication/imaging (incl. earth observation and remote sensing)  Virtual/augmented reality  Cellular networks  Smartphone applications  Text message communications  Bluetooth communications  5G  Smartphones and tablet computing devices  Internet of things (IoT)  Smart city infrastructure  Wireless sensor networks  Biosensors  Digital DNA/RNA/protein analysis  Lab-on-chip (LOC)  Nanotechnology  Online learning platforms  Online self-assessment tools  Information/resource management tools (incl.dashboards)  Quantum computing  Mathematical models/simulations  Micro-blogging platforms  Blogging platforms  Instant messaging platforms  Networking platforms  Software elaboration platforms  Photo/video-sharing platforms  Wearables (incl. smart fabrics, ingestibles) | | Advanced manufacturing technologies  Autonomous devices and systems  Blockchain / Distributed ledger technology  Cloud computing / cloud-based networks  Cognitive technologies  Crowdsourcing platforms  Data analytics (including big data)  eHealth  Imaging and sensing technologies (including GIS)  Immersive technologies  Integrated and ubiquitous fixed and mobile networks  Internet of things (IoT)  Nanotechnology and microsystems  Online tools and platforms  Quantum computing  Simulation  Social media platforms  Wearables (including ingestibles)  NA | | 3D-printing  Drones  Robotics  Blockchain / Distributed ledger technology  Cloud computing / cloud-based networks  Artificial intelligence (AI)  Expert systems  Facial recognition  Machine learning (ML)  Natural language processing  (Artificial) neural networks  Crowdsourcing  Data mining  Data analytics  Big data  Bioinformatics  Parallel computing  Social media and mobile data analysis  Digital health /eHealth / mHealth  Electronic health records (EHRs)  Telemedicine  Geographic information systems (GIS)  Health informatics  Image processing  Infrared sensing  Satellite communication/imaging (incl. earth observation and remote sensing)  Virtual/augmented reality  Cellular networks  Smartphone applications  Text message communications  Bluetooth communications  5G  Smartphones and tablet computing devices  Internet of things (IoT)  Smart city infrastructure  Wireless sensor networks  Biosensors  Digital DNA/RNA/protein analysis  Lab-on-chip (LOC)  Nanotechnology  Online learning platforms  Online self-assessment tools  Information/resource management tools (incl.dashboards)  Quantum computing  Mathematical models/simulations  Micro-blogging platforms  Blogging platforms  Instant messaging platforms  Networking platforms  Software elaboration platforms  Photo/video-sharing platforms  Wearables (incl. smart fabrics, ingestibles)  NA | |
|  |  | |  |  |  |  |

| Public health key function(s) impacted 1 | Public health key function(s) impacted 2 | Obstacle/barrier type |
| --- | --- | --- |
| Screening and diagnostics  Surveillance and monitoring  Forecasting  Signal/outbreak detection and validation  Outbreak response  Communication and collaboration  Contact tracing | Screening and diagnostics  Surveillance and monitoring  Forecasting  Signal/outbreak detection and validation  Outbreak response  Communication and collaboration  Contact tracing  N/A | Data  Resources and costs (including human resources)  Network infrastructure  Physical infrastructure  Safety  Ethical  Legal/regulatory  Political  Environmental  Social  Technical  Not reported |

Table A4-2. Extraction template and drop-down menus for the review of nonacademic literature (review time frame: January 1, 2020, to October 13, 2020).

| Article type | Study type | Is it a comparative study? | Geographical context of implementation (EU/EEA, Non-EU/EEA or Both) | Specific country context | Contributing Region (EU/EEA, Non-EU/EEA or Both - Geographical context of technology development) | Prominent contributing countries  (Geographical context of technology development) |
| --- | --- | --- | --- | --- | --- | --- |
| News article/ press release/ blog post | N/A | Yes  No  N/A | EU/EEA  Non-EU/EEA  Both  Not reported | Global  Afghanistan  Albania  Algeria  Andorra  Angola  Antigua and Barbuda  Argentina  Armenia  Australia  Austria  Azerbaijan  Bahamas  Bahrain  Bangladesh  Barbados  Belarus  Belgium  Belize  Benin  Bhutan  Bolivia  Bosnia and Herzegovina  Botswana  Brazil  Brunei  Bulgaria  Burkina Faso  Burundi  Côte d'Ivoire  Cabo Verde  Cambodia  Cameroon  Canada  Central African Republic  Chad  Chile  China  Colombia  Comoros  Congo (Congo-Brazzaville)  Costa Rica  Croatia  Cuba  Cyprus  Czechia (Czech Republic)  Democratic Republic of the Congo  Denmark  Djibouti  Dominica  Dominican Republic  Ecuador  Egypt  El Salvador  Equatorial Guinea  Eritrea  Estonia  Eswatini  Ethiopia  Fiji  Finland  France  Gabon  Gambia  Georgia  Germany  Ghana  Greece  Grenada  Guatemala  Guinea  Guinea-Bissau  Guyana  Haiti  Holy See  Honduras  Hungary  Iceland  India  Indonesia  Iran  Iraq  Ireland  Israel  Italy  Jamaica  Japan  Jordan  Kazakhstan  Kenya  Kiribati  Kuwait  Kyrgyzstan  Laos  Latvia  Lebanon  Lesotho  Liberia  Libya  Liechtenstein  Lithuania  Luxembourg  Madagascar  Malawi  Malaysia  Maldives  Mali  Malta  Marshall Islands  Mauritania  Mauritius  Mexico  Micronesia  Moldova  Monaco  Mongolia  Montenegro  Morocco  Mozambique  Myanmar  Namibia  Nauru  Nepal  Netherlands  New Zealand  Nicaragua  Niger  Nigeria  North Korea  North Macedonia  Norway  Oman  Pakistan  Palau  Palestine State  Panama  Papua New Guinea  Paraguay  Peru  Philippines  Poland  Portugal  Qatar  Romania  Russia  Rwanda  Saint Kitts and Nevis  Saint Lucia  Saint Vincent and the Grenadines  Samoa  San Marino  Sao Tome and Principe  Saudi Arabia  Senegal  Serbia  Seychelles  Sierra Leone  Singapore  Slovakia  Slovenia  Solomon Islands  Somalia  South Africa  South Korea  South Sudan  Spain  Sri Lanka  Sudan  Suriname  Sweden  Switzerland  Syria  Tajikistan  Tanzania  Thailand  Timor-Leste  Togo  Tonga  Trinidad and Tobago  Tunisia  Turkey  Turkmenistan  Tuvalu  Uganda  Ukraine  United Arab Emirates  United Kingdom  United States of America  Uruguay  Uzbekistan  Vanuatu  Venezuela  Vietnam  Yemen  Zambia  Zimbabwe  Not reported  Taiwan  Hong Kong | EU/EEA  Non-EU/EEA  Both  Not reported | Afghanistan  Albania  Algeria  Andorra  Angola  Antigua and Barbuda  Argentina  Armenia  Australia  Austria  Azerbaijan  Bahamas  Bahrain  Bangladesh  Barbados  Belarus  Belgium  Belize  Benin  Bhutan  Bolivia  Bosnia and Herzegovina  Botswana  Brazil  Brunei  Bulgaria  Burkina Faso  Burundi  Côte d'Ivoire  Cabo Verde  Cambodia  Cameroon  Canada  Central African Republic  Chad  Chile  China  Colombia  Comoros  Congo (Congo-Brazzaville)  Costa Rica  Croatia  Cuba  Cyprus  Czechia (Czech Republic)  Democratic Republic of the Congo  Denmark  Djibouti  Dominica  Dominican Republic  Ecuador  Egypt  El Salvador  Equatorial Guinea  Eritrea  Estonia  Eswatini  Ethiopia  Fiji  Finland  France  Gabon  Gambia  Georgia  Germany  Ghana  Greece  Grenada  Guatemala  Guinea  Guinea-Bissau  Guyana  Haiti  Holy See  Honduras  Hungary  Iceland  India  Indonesia  Iran  Iraq  Ireland  Israel  Italy  Jamaica  Japan  Jordan  Hong Kong  Kazakhstan  Kenya  Kiribati  Kuwait  Kyrgyzstan  Laos  Latvia  Lebanon  Lesotho  Liberia  Libya  Liechtenstein  Lithuania  Luxembourg  Madagascar  Malawi  Malaysia  Maldives  Mali  Malta  Marshall Islands  Mauritania  Mauritius  Mexico  Micronesia  Moldova  Monaco  Mongolia  Montenegro  Morocco  Mozambique  Myanmar  Namibia  Nauru  Nepal  Netherlands  New Zealand  Nicaragua  Niger  Nigeria  North Korea  North Macedonia  Norway  Oman  Pakistan  Palau  Palestine State  Panama  Papua New Guinea  Paraguay  Peru  Philippines  Poland  Portugal  Qatar  Romania  Russia  Rwanda  Saint Kitts and Nevis  Saint Lucia  Saint Vincent and the Grenadines  Samoa  San Marino  Sao Tome and Principe  Saudi Arabia  Senegal  Serbia  Seychelles  Sierra Leone  Singapore  Slovakia  Slovenia  Solomon Islands  Somalia  South Africa  South Korea  South Sudan  Spain  Sri Lanka  Sudan  Suriname  Sweden  Switzerland  Syria  Taiwan  Tajikistan  Tanzania  Thailand  Timor-Leste  Togo  Tonga  Trinidad and Tobago  Tunisia  Turkey  Turkmenistan  Tuvalu  Uganda  Ukraine  United Arab Emirates  United Kingdom  United States of America  Uruguay  Uzbekistan  Vanuatu  Venezuela  Vietnam  Yemen  Zambia  Zimbabwe  UN agency  International organisation  Not reported |

| High-level technology group 1 | Specific digital technology 1 | High-level technology group 2 | Specific digital technology 2 |
| --- | --- | --- | --- |
| Advanced manufacturing technologies  Autonomous devices and systems  Blockchain / Distributed ledger technology  Cloud computing / cloud-based networks  Cognitive technologies  Crowdsourcing platforms  Data analytics (including Big Data)  eHealth  Imaging and sensing technologies (including GIS)  Immersive technologies  Integrated and ubiquitous fixed and mobile networks  Online platforms  Social media  Internet of things (IoT)  Nanotechnology and microsystems  Quantum computing  Simulation  Wearables (including ingestibles) | 3D-printing  Drones  Robotics  Blockchain / Distributed ledger technology  Cloud computing / cloud-based networks  Artificial intelligence (AI)  Expert systems  Machine learning (ML)  Natural language processing  Facial recognition  (Artificial) neural networks  Crowdsourcing  Data mining  Data analytics  Big Data  Health informatics  Parallel computing  Social media and mobile data analysis  Digital health /eHealth / mHealth  Electronic health records (EHRs)  Telemedicine  Geographic Information Systems (GIS)  Health informatics  Image processing  Infrared sensing  Satellite communication/imaging (incl. earth observation and remote sensing)  Virtual/augmented reality  Cellular networks  Text message communications  Smartphone applications  Bluetooth  Smartphones and tablet computing devices  Online learning platforms  Online self-assessment tools  Information management tools  Micro-blogging platforms  Blogging platforms  Instant messaging platforms  Networking platforms  Software elaboration platforms  Photo/video-sharing platforms  Internet of things (IoT)  Wireless sensor networks  Biosensors  Digital DNA/RNA/protein analysis  Lab-on-chip (LOC)  Nanotechnology  Quantum computing  Mathematical models/simulations  Wearables (incl. smart fabrics, ingestibles) | Advanced manufacturing technologies  Autonomous devices and systems  Blockchain / Distributed ledger technology  Cloud computing / cloud-based networks  Cognitive technologies  Crowdsourcing platforms  Data analytics (including Big Data)  eHealth  Imaging and sensing technologies (including GIS)  Immersive technologies  Integrated and ubiquitous fixed and mobile networks  Online platforms  Social media  Internet of things (IoT)  Nanotechnology and microsystems  Quantum computing  Simulation  Wearables (including ingestibles) | 3D-printing  Drones  Robotics  Blockchain / Distributed ledger technology  Cloud computing / cloud-based networks  Artificial intelligence (AI)  Expert systems  Machine learning (ML)  Natural language processing  Facial recognition  (Artificial) neural networks  Crowdsourcing  Data mining  Data analytics  Big Data  Health informatics  Parallel computing  Social media and mobile data analysis  Digital health /eHealth / mHealth  Electronic health records (EHRs)  Telemedicine  Geographic Information Systems (GIS)  Health informatics  Image processing  Infrared sensing  Satellite communication/imaging (incl. earth observation and remote sensing)  Virtual/augmented reality  Cellular networks  Text message communications  Smartphone applications  Bluetooth  Smartphones and tablet computing devices  Online learning platforms  Online self-assessment tools  Information management tools  Micro-blogging platforms  Blogging platforms  Instant messaging platforms  Networking platforms  Software elaboration platforms  Photo/video-sharing platforms  Internet of things (IoT)  Wireless sensor networks  Biosensors  Digital DNA/RNA/protein analysis  Lab-on-chip (LOC)  Nanotechnology  Quantum computing  Mathematical models/simulations  Wearables (incl. smart fabrics, ingestibles) |

| Public health key function(s) impacted 1 | Public health key function(s) impacted 2 | Obstacle/barrier type |
| --- | --- | --- |
| Screening and diagnostics  Surveillance and monitoring  Forecasting  Signal/outbreak detection and validation  Outbreak response  Communication and collaboration  Contact tracing | Screening and diagnostics  Surveillance and monitoring  Forecasting  Signal/outbreak detection and validation  Outbreak response  Communication and collaboration  Contact tracing  N/A | Data  Resources and costs (including human resources)  Network infrastructure  Physical infrastructure  Safety  Ethical  Legal/regulatory  Political  Environmental  Social  Technical  Not reported |
