# Supplementary material for: Assessing the Implementation of Digital Innovations in Response to the COVID-19 Pandemic to Address Key Public Health Functions: Scoping Review of Academic and Nonacademic Literature
Source: JMIR Public Health Surveill. 2022 Jul 6;8(7):e34605. doi: 10.2196/34605 (PMC9301563; doi:10.2196/34605)
Supplement: Multimedia Appendix 5 [file publichealth_v8i7e34605_app5.docx]

# Appendix 5: Cross-analysis tables

Table A5-1. Mapping table showing the number of digital innovations employing each high-level technology group (shown along the x-axis) in each country (shown along the y-axis) in the review of academic literature (review time frame: January 1, 2020, to September 15, 2020).


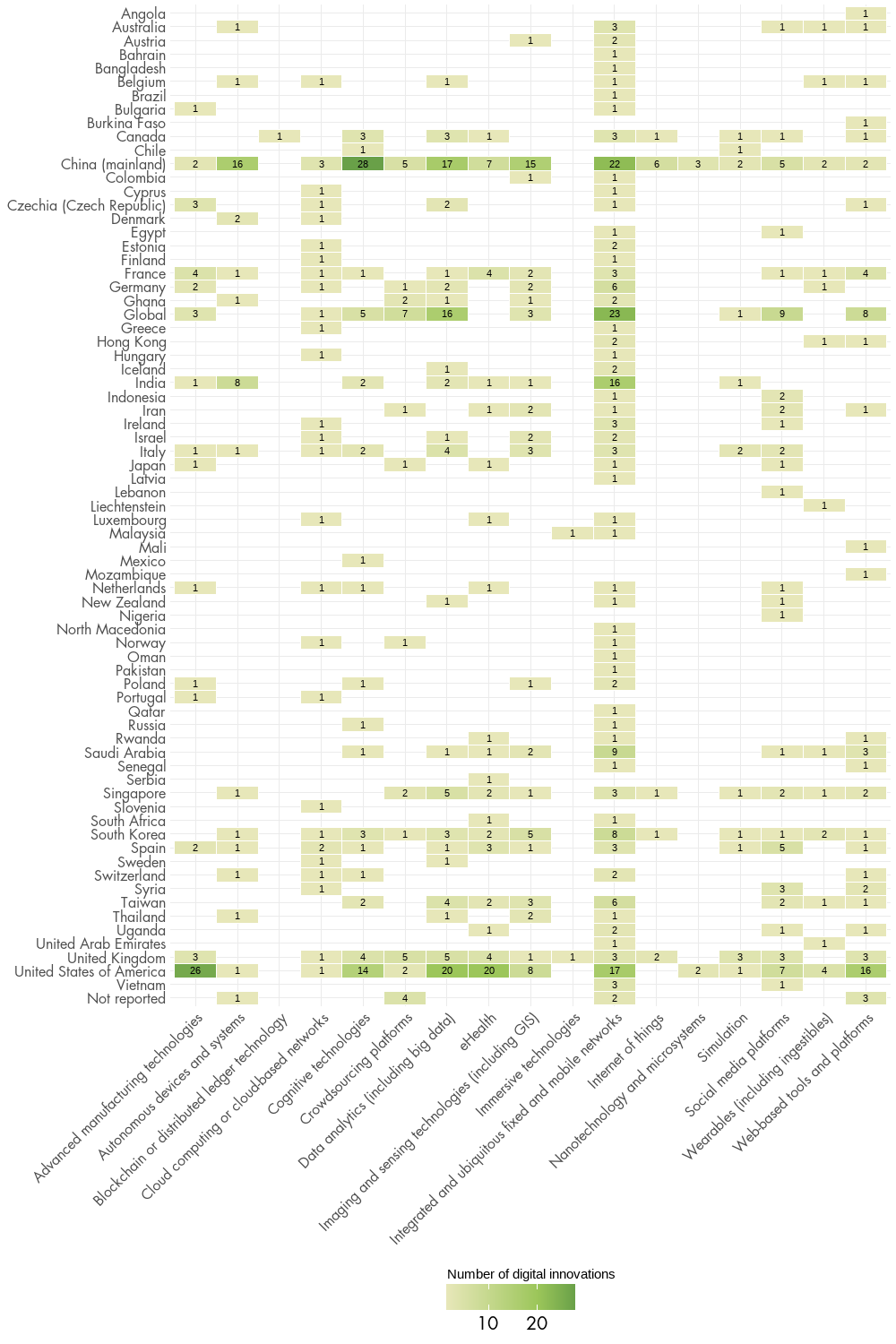


Source: RAND Europe

Table A5-2: Mapping table showing the number of digital innovations employing each high-level technology group (shown along the x-axis) in each country (shown along the y-axis) in the review of nonacademic literature (review time frame: January 1, 2020, to October 13, 2020).


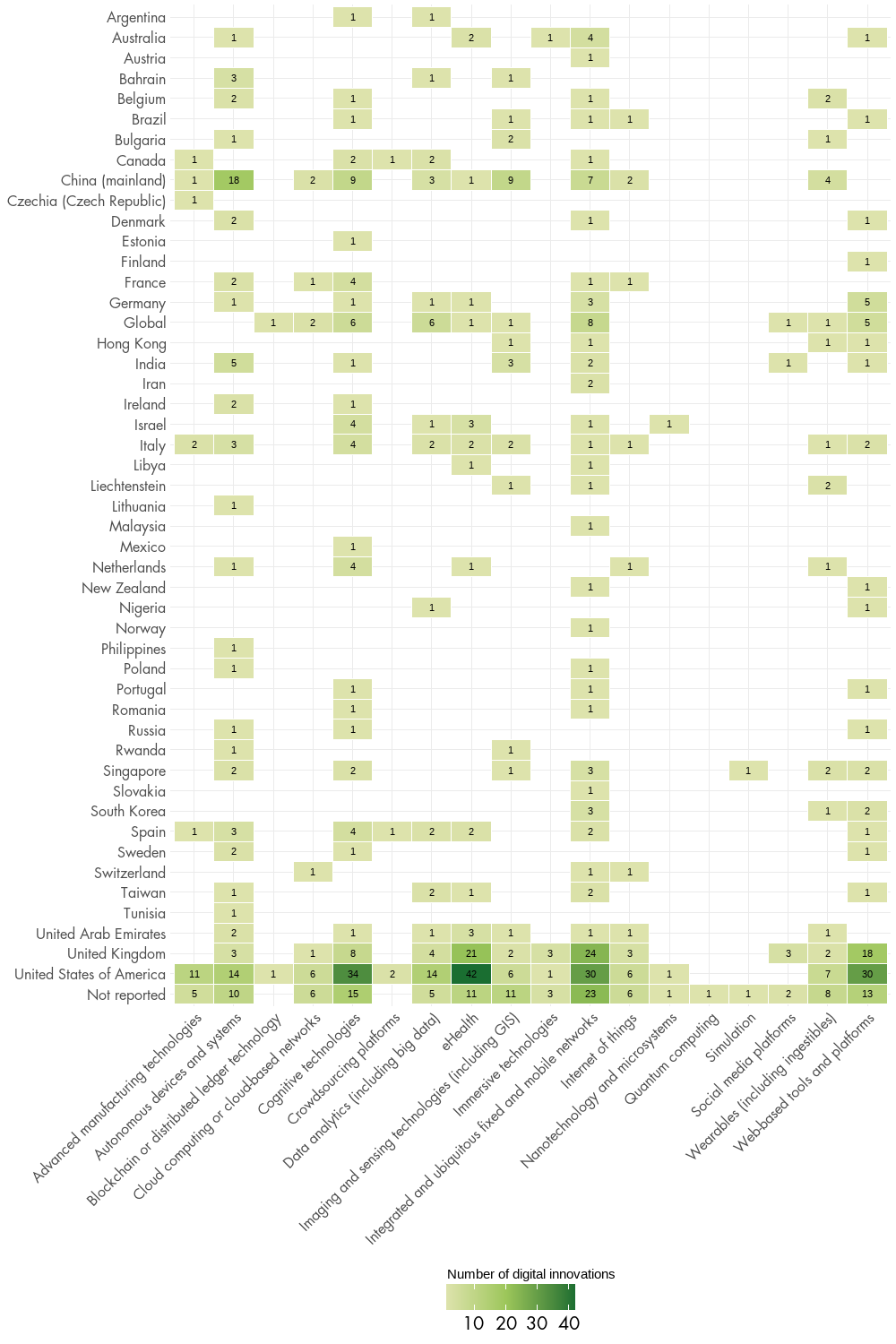


Source: RAND Europe

Table A5-3. Number of digital innovations employing each high-level technology group for each public health key function in the review of academic literature (review time frame: January 1, 2020, to September 15, 2020).


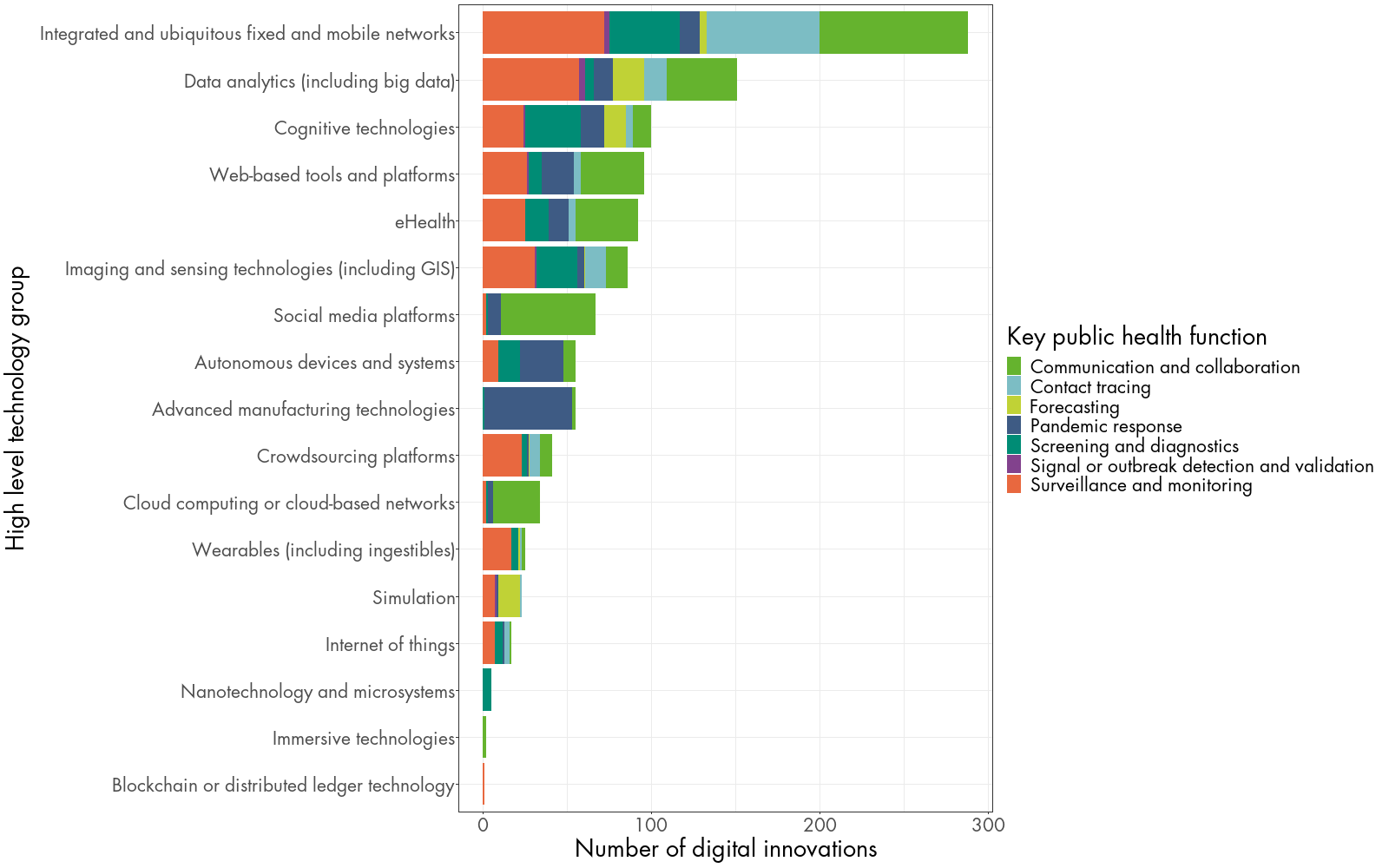


Source: RAND Europe

Table A5-4: Number of digital innovations employing each high-level digital technology group for each public health key function in the review of nonacademic literature (review time frame: January 1, 2020, to October 13, 2020).


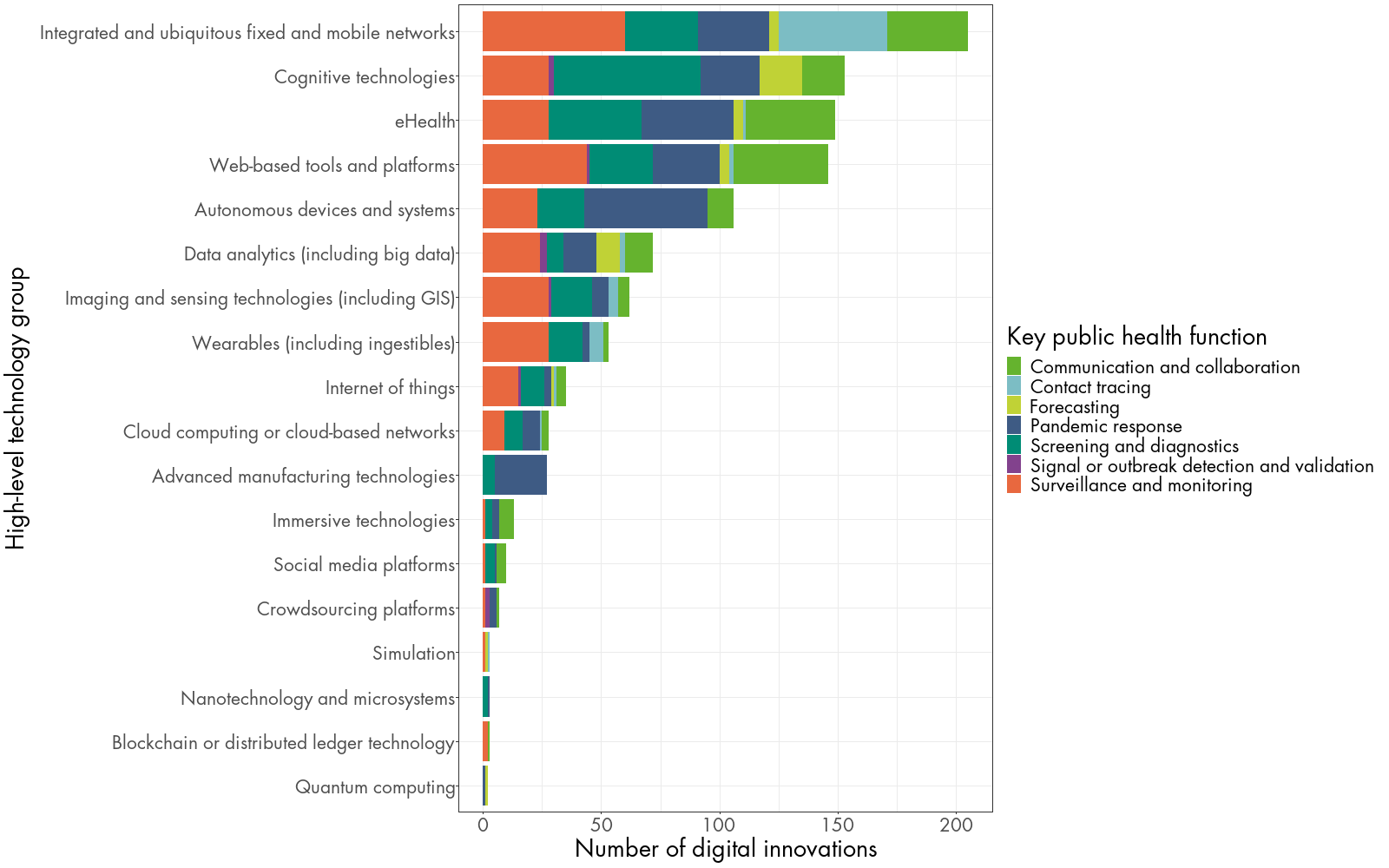


Source: RAND Europe
